# Identifying delays impacting maternal and perinatal deaths using a facility-based death audit review system integrated with community engagement: a mixed methods study

**DOI:** 10.7189/jogh.16.04117

**Published:** 2026-04-24

**Authors:** Zahid Memon, Wardah Ahmed, Shah Muhammad, Sajid Soofi, Shanti Chouhan, Arjumad Rizvi, Paul Barach, Zulfiqar A Bhutta

**Affiliations:** 1 Aga Khan University, Department of Community Health Sciences, Karachi, Pakistan; 2 Aga Khan University, Center of Excellence in Women and Child Health, Karachi, Pakistan; 3 Aga Khan University, Institute of Global Health and Development, Karachi, Pakistan; 4 Thomas Jefferson Univeristy, Jefferson College of Population Health, Philadelphia, USA; 5 University of North Carolina, Sheps Center for Health Outcomes, Chapel Hill, USA; 6 Imperial College London, School of Public Health, London, UK

## Abstract

**Background:**

Pakistan’s persistently high maternal (178 per 100 000 livebirths) and neonatal (41 per 1000 livebirths) mortality ratios are compounded by lack of reliable data on the root causes and preventable risk factors, which hinders their effective use in improving care and progress toward safe, high-quality services. We sought to identify system delays impacting maternal and perinatal deaths using a facility-based death audit review system integrated with community engagement for implementing actionable solutions.

**Methods:**

We used a mixed methods, concurrent parallel study design at three secondary level healthcare facilities in District Matiari, Sindh, Pakistan. We reviewed 319 cases that included 19 maternal deaths (MDs), 185 neonatal deaths (NDs) and 115 cases of stillbirth (SB) using the WHO based death audit review system integrated with community engagement. We documented quantitative data for all cases using descriptive statistics and simultaneously collected qualitative narratives, which we then analysed and categorised across the four delays model. Audit committees comprising facility staff and community representatives met quarterly to review cases.

**Results:**

We observed delay patterns across death types. MDs were predominantly influenced by delays 3 (reaching facility: 74%) and 4 (receiving adequate care: 74%), reflecting system-level barriers in access and quality of care. SB cases were primarily associated with delays 1 (recognition of danger signs: 64%) and 2 (decision to seek care: 60%), highlighting household-level knowledge and decision-making gaps. NDs were primarily affected by delays 4 (receiving adequate care: 54%) and 1 (recognition of danger signs: 48%), indicating both facility capacity constraints and early recognition failures. The analysis revealed four interconnected themes explaining these delays: lack of education and awareness (delays 1 and 2), inadequate transport mechanisms (delay 3), multiple referrals (overlapping delays 3 and 4), and limited facility operational hours and delayed medical care (delay 4).

**Conclusions:**

The four delays model identified patterns of preventable factors contributing to maternal and perinatal deaths across household and health system levels. Health systems need to invest in women’s access to, and the availability of, healthcare facilities both during and after pregnancy. Scaling up and implementing audit review systems with learning feedback loops is key to systematically identifying and contributing to addressing preventable delays in resource constrained settings, particularly when comprehensive national-level mortality data are lacking.

Pakistan faces alarmingly high rates of maternal and perinatal deaths with maternal mortality ratios of 178 per 100 000 live births and neonatal mortality ratios of 41 per 1000 live births [1]. The global targets set by the United Nations Sustainable Development Goals for 2030 aim to reduce the former to under 70 per 100 000 live births and the latter to 12 per 1000 live births [2]. However, progress has been limited in Pakistan and many low- and middle-income countries (LMICs) [3]. There is also a lack of reliable data on the root causes and contributing risk factors for mortality that hinders safe and high-quality delivery in LMICs. Accurate, timely data is essential for understanding the factors contributing to maternal deaths (MDs), neonatal deaths (NDs), and stillbirths (SBs), and can thus inform targeted interventions to reduce mortality rates in Pakistan [4–6].

The implementation of maternal and perinatal mortality audits has globally emerged as a crucial strategy for improving healthcare quality and supporting the effective implementation of Sustainable Development Goals related to maternal and perinatal health [7]. Several systematic reviews of maternal and perinatal mortality audits identified preventable factors contributing to maternal and neonatal deaths, with audits being shown to reduce perinatal mortality by 30% (95% confidence interval = 21–38%), and by identifying care deficiencies and implementing corrective measures [7–10] (**Box 1**).

Box 1 **Previous evidence and added values**

**What is already known on this topic**

Facility audit systems like the Maternal & Perinatal Death Surveillance and Response have been shown to successfully improve the reporting, recording, tracking, and auditing of maternal and child deaths. As part of ongoing efforts, the WHO has implemented audit mechanisms in Khyber Pakhtunkhwa and Baluchistan provinces, while UNICEF has supported the implementation of similar systems in Sindh province. Challenges, however, persist regarding the efficacy and implementation of mortality reviews: recommendations from reviews are inconsistently recorded and implemented, which hinders uniform and effective actions to address locally known and globally established root causes for maternal and child mortality.

**What this study adds**

This is an implementation study of a death audit review system (audit cycle) at secondary-level care health facilities in a rural district of Pakistan, based on WHO guidelines. A distinct feature was co-design of the doable solutions the by active engagement of local community representatives. The four delays model offers a clear framework for understanding barriers to maternal and neonatal mortality in LMICs like Pakistan, focusing on the delays in recognising danger signs, decision in seeking care, reaching facilities, and receiving appropriate treatment at the facility. Our study provides an empirical foundation for 24/7 staffing, referral coordination, and integrating community members, healthcare providers, and policymakers to identify preventable factors and barriers contributing to maternal and perinatal deaths. Our results underscore the implementation of doable solutions to strengthen health systems, particularly in less developed regions where comprehensive national-level mortality data are lacking, and mortality continues to be high. Findings are likely transferable to similar rural LMIC settings.

Countries utilising systematic quality audits report higher maternal and perinatal mortality ratios, reflecting improved and earlier visibility of maternal health issues and enhanced intervention planning [11]. However, barriers remain in terms of low numbers of trained community healthcare personnel and ongoing challenges of learning from and integrating audit findings into routine practice to ensure actionable outcomes. The under-utilisation of death review audit systems in Pakistan, for example, highlights an urgent need for such systematic implementation and evaluation [12,13].

The conceptual framework underlying this study is grounded in the three delays model introduced by Thaddeus and Maine in 1994, which has been pivotal to elucidating the factors contributing to pregnancy-related mortality [14]. This model identifies three critical delays: in deciding to seek care, in reaching a health facility, and in receiving adequate care at the facility [14–16]. Recognising the complexities of maternal and newborn health in low-resource settings, subsequent research has expanded this framework into the four delays model (FDM), which divides the first delay into two distinct categories to better capture individual (household) level barriers [17–19].

In this study, we used the FDM, rather than the original three-delay model for several context-specific reasons. First, previous research in Pakistan has demonstrated that recognition of danger signs and decision-making, as captured in the first delay, represent distinct barriers requiring different interventions: recognition required health education and awareness interventions, while decision-making requires addressing gender dynamics and financial access [17,20]. Second, the distinction between delays 1 and 2 allows for more targeted identification of whether interventions should focus on community health education or empowering women and addressing household decision-making structures. Third, emerging evidence suggests that the subdivision of home delays better captures the reality in patriarchal societies where women may recognise danger, but lack autonomy to act [21–23]. This framework was applied in similar settings and recommended by World Health Organization (WHO) for maternal death surveillance and response systems [18,19]. Our objective was to identify and categorise the delays contributing to maternal and perinatal deaths using FDM through facility-based deaths audit review system.

We hypothesised that applying the FDM could identify the preventable factors impacting maternal and perinatal deaths in Pakistan. We further posited that a process linking and engaging community representatives using a facility-based death audit review system could complete feedback loops [23] and prevent future delays especially around early recognition and rapid care seeking.

## METHODS

**Figure 1:**
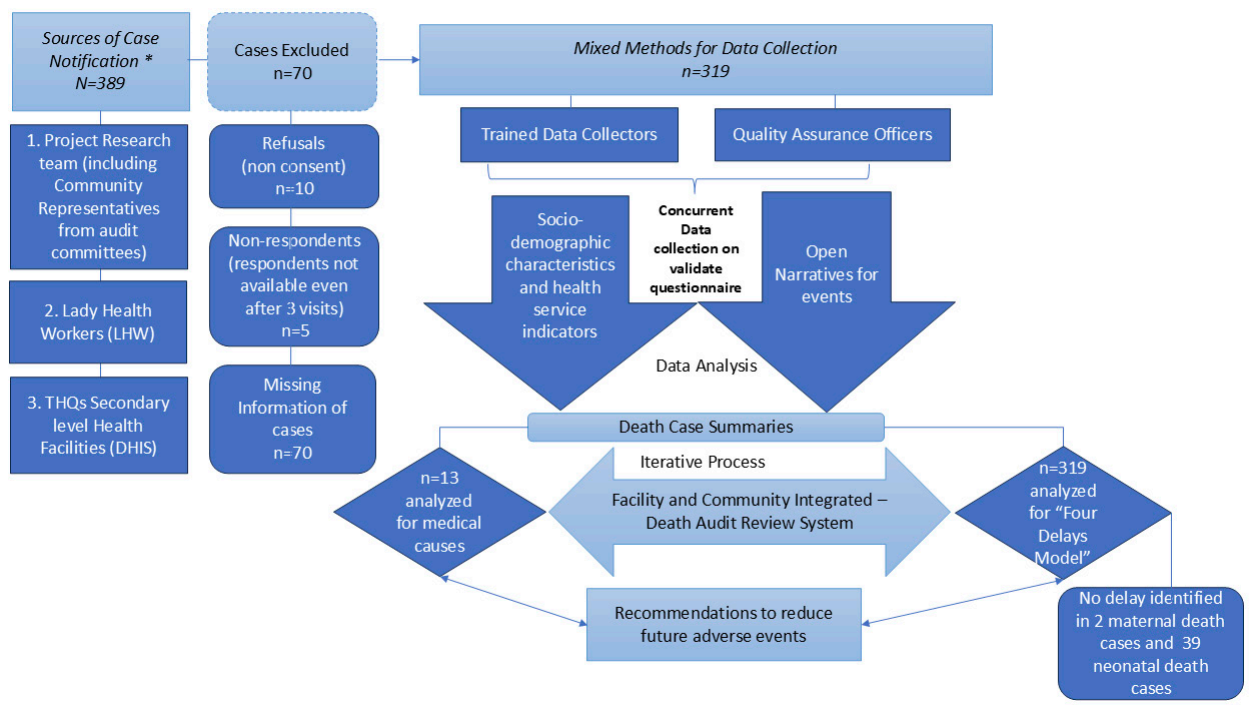
Case flow diagram showing sources of death notifications, reasons of exclusion, and audit committee review process.

Our previously published protocol details the study phases [24]. This mixed-methods study adopted a concurrent parallel design, where quantitative audit data and qualitative interview data were collected concurrently and integrated during the analysis phase (**Figure 1**), and was delivered in District Matiari from January 2022 to April 2023 (16 months). We report our findings per the GRAMMS checklist [25] (**Online Supplementary Document**). Quantitative and qualitative strands were analysed separately and then integrated using triangulation during interpretation.

For this study, we adopted the following operational definitions of FDM. Delay 1 (recognition of danger signs) was defined as a delay in recognising the severity of the illness and identifying danger signs by the woman, family members or community members. This includes prolonged labour, convulsion, high grade fever, or foetal distress that require urgent medical attention. This delay 1 split and distinction is particularly relevant in patriarchal contexts where recognition does not necessarily translate into agency. Delay 2 (decision making to seek care) was understood as a delay in deciding to seek care once danger signs are recognised. This encompasses barriers such as need for permission from family members (particularly male household heads), financial constraints, cultural beliefs, or previous negative healthcare experiences that impede the decision to seek facility-based care. Delay 3 (reaching facility) was defined as a delay in reaching an appropriate health facility due to transportation challenges, distance, cost of transport, poor road conditions, or difficulties in accessing ambulance services. This also includes delays related to multiple transfers between facilities before reaching appropriate care. Lastly, delay 4 (receiving adequate treatment) was seen as a delay in receiving adequate and timely care once at the health facility. This includes delays in assessment, diagnosis, treatment initiation, surgical interventions, blood transfusion, or referral to high-level facilities, as well as gaps in staff availability, supplies, or equipment.

### Study setting

The Pakistan Bureau of Statistics’ 2023 report estimates the population of Pakistan to be approximately 241.5 million within four provinces (Sindh, Punjab, Balochistan, and Khyberpakhtunkhwah). The Sindh province is estimated to have around 47.9 million people [26]. Matiari District, with a population of 850 000, is one of the 30 districts of Sindh Province, located 185 km from provincial capital Karachi. It is divided into three (*taluka*) sub-districts (Hala, Saeedabad, and Matiari) comprising 30 union councils and 112 villages.

Matiari District’s healthcare infrastructure includes 20 basic health units (BHUs); 4 rural health centres (RHCs); 3 *taluka* headquarter hospitals (THQ), now upgraded to district headquarter hospital (Hala, Saeedabad, and Matiari); and 23 dispensaries. The healthcare facilities are managed by the Directorate General Health Services of the Government of Sindh. The District’s combined capacity of government and private health facilities is 422 beds. In terms of healthcare personnel, the District hosts one surgeon, 3 gynaecologists, 4 paediatricians, 210 general medical officers, 11 nurses, and 9 lady health visitors, who are supported by 429 lady health workers (LHWs), *i.e.* community outreach health workers, overseen by 17 lady health supervisors (heath facility-based) across district. There are also 28 community midwives and 90 other paramedical staff members. The district has one government blood bank.

### Facility-based death audit review system and integrating community engagement

The death audit review system comprises six phases:

1. identifying facility and community leadership through consultative meetings with government district health offices;

2. establishing the audit committee (including healthcare providers, community representatives and district health officials) under the supervision of the district health officer;

3. initiating death audits and case reviews with ongoing community engagement (verbal autopsy);

4. training the audit committee members and data collection team;

5. implementing the WHO iterative audit cycle (monthly meetings) [8–10], with a component of sharing findings with community and district health population management teams;

6. conducting quarterly reviews and refresher training.

More details can be found in our published protocol [24].

### Study population and demographic coverage

The three study facilities (THQ Hala, THQ Saeedabad, and THQ Matiari) are secondary-level health facilities serving the population of District Matiari. Each THQ serves as referral centre for BHUs and RHCs in its catchment area. The combined catchment area of the three THQs, based on 2023 census data [26], encompasses approximately 480 000 population. Using Pakistan’s crude birth rate of 27.2 per 1000 population, we estimated 13 056 annual births in this combine catchment area, or approximately 17 405 births over 16-month study period. A total of 15 654 deliveries were recorded in the LHW Management Information System for Matiari District during the study period.

### Death notification sources and study variables

We identified MDs, SBs, and NDs from three sources: the research team, including community representatives from audit committees; LHW-MIS; and THQs secondary-level health facilities, notified in the district health information system. We used a case notification form for audit data (**Online Supplementary Document**), while the data collection team visited the households of the deceased to gather detailed information from family using a questionnaire-case review form (**Online Supplementary Document**). The study variables included the sources of case notification and health service indicators, such as the place of antenatal care (ANC), place of death, time of death, and the number of referrals for all notified cases. Additionally, we recorded the sociodemographic characteristics of the cases reviewed by the audit committees (**Online Supplementary Document**). We collected narratives from mothers and/or families through interviews and subsequently analysed them using qualitative thematic analysis to identify key themes and contributing factors to mortality.

### Data collection

We collected quantitative and qualitative narrative data using the WHO death audit tool for health facility audits [9], which was adapted, translated, and piloted for the community notification and reporting of deaths (available by request). A verbal autopsy approach based on open narratives was employed for data collection from the community [27,28]. Trained data collectors conducted in-person interviews under the supervision of one co-author (SC) in the local Sindhi language. The detailed written interviews with mother/or family members were conducted 1–12 weeks following the maternal or perinatal death, lasted 30–60 minutes each, and were later transcribed and translated into English. The data collectors were proficient in Sindhi language and knowledgeable about local customs and culture; they used a series of prompts and piloted questions to gather detailed, first-hand information about the events leading to maternal deaths, stillbirths, and neonatal deaths. The interviews focused on understanding the adverse event (death) and contributing factors related to maternal and perinatal deaths in the study setting [9,27,28]. The quality assurance of the data collection was rigorously assessed the reliability of the data, its availability, and its completeness by three researchers (SC, WA, SM) before inclusion in the analysis and subsequent categorisation under the FDM.

We implemented a systematic duplicate detection across notification channels using a three-step process:

− automated matching based on mother’s name, father’s/husband’s name, village/address, and approximate date of death in the data file;

− manual review of potential matches (17 potential duplicates were identified) and removed from the datasets;

− confirmation through comparison of detailed information from all notifications.

### Death case reviews and quality assurance

We developed the summaries of each death case for case reviews and analysis from the quantitative and qualitative narrative data. All death cases were reviewed by the audit committee members, including three gynaecologists, one paediatrician, community representatives, district health officers, and two district managers assure that all steps were followed during the meetings. Drawing on the FDM, audit committee members provided actionable recommendations about respective health facilities and in their catchment area. We employed a rigorous two-step double data entry and random checks to ensure data accuracy and completeness. Two data collectors independently entered all records into the excel sheet, after which the co-author SC verified the consistency between the entered data from primary audit forms.

### Ethics and informed consent

The study received ethical approval from the Aga Khan University (2021-6426-19638) on 29 November 2021. The primary respondents were mothers (in cases of SBs and NDs) and immediate relatives, such as spouses or parents (interviewed for MDs). We obtained written informed consent from all respondents before the interviews. The consent script was read aloud and their consent was recorded on the form in the local language. The consent process varied depending on the respondent's age. For married women aged ≥18 years, we obtained consent directly from the respondent. For married adolescents aged 15–17 years . (12 participants, all mothers who had experienced SBs or NDs), consent was obtained from the head of the household or husband, along with assent from the respondent in the presence of a witness. Under Pakistan's legal framework and Aga Khan University ERC requirements, married adolescents are considered emancipated minors, but still require guardian consent to participate in research. We did not conduct interviews if a guardian consented, but the adolescent mother declined assent (two cases). If the adolescent wished to participate, but the guardian declined, we respected the guardian’s refusal (all guardians who were approached consented). The interview proceeded after permission was granted; otherwise, the process was terminated.

All interviews were conducted in private settings where guardians could not overhear, typically in separate rooms or outdoor spaces within the household boundary. Guardians and other family members were explicitly asked to leave the interview space, unless respondent asked to be accompanied. All interviews were conducted by female data collectors of similar age and background to reduce intimidation and facilitate disclosure. Interviewers checked agreement to interview multiple times during interview (‘Are you comfortable continuing?’) and watched for non-verbal cues of distress or coercion. This structured and culturally sensitive consent process ensured ethical compliance and the respectful participation of all respondents [24].

### Data analysis

#### Quantitative analysis

We used Stata, version 17 (StataCorp LLC., College Station, Texas, USA) to calculate the frequencies and percentages for health services indicators, sociodemographic indicators, and the distribution of death cases among the four delays. We reported the mean and standard deviation for gravidity, parity, age at time of first pregnancy, and inter pregnancy interval.

#### Qualitative analysis

We used a deductive thematic analysis approach [29] guided by the FDM conceptual framework [17]. This involved the systematic organisation, categorisation, and summarisation of qualitative narratives and other variables to identify any underlying patterns and trends. According to the FDM, we devised codes and allocated them into subthemes and themes, where we identified multifaceted, overlapping delays affecting healthcare access and delivery. This coding was done by two independent coders – a research associate with training in qualitative methods who was fluent in Sindhi and English language (KK) and a senior researcher with training in qualitative methods and expert in English language (FS). The coders independently read all 319 verbal autopsy narratives and coded each case for presence/absence of each delay type. Coding decisions were recorded in a structured Excel database with fields for each delay type, specific evidence from narrative supporting the coding decision, relevant quotes, and coder comments/uncertainties. An epidemiologist with mixed-methods expertise (SM) and a statistician (AR) provided third-party adjudication for discrepancies. This approach ensured that the analysis was aligned with the theoretical framework, thus enhancing the rigor and relevance of the findings.

We acknowledge that quote selection involves researcher interpretation and choices that can shape narrative. To mitigate bias, we selected quotes as a team, rather than through individual decision, and we prioritised quotes that illustrated patterns seen across multiple cases, rather than unique perspectives; that presented both family and health system perspectives, rather than solely attributing blame to one side; and that illustrated complexity and ambiguity (*e.g.* cases where delays were multi factorial), rather than only clear-cut examples. We did not calculate inter-coder reliability statistics; discrepancies were resolved through consensus.

## RESULTS

**Figure 2:**
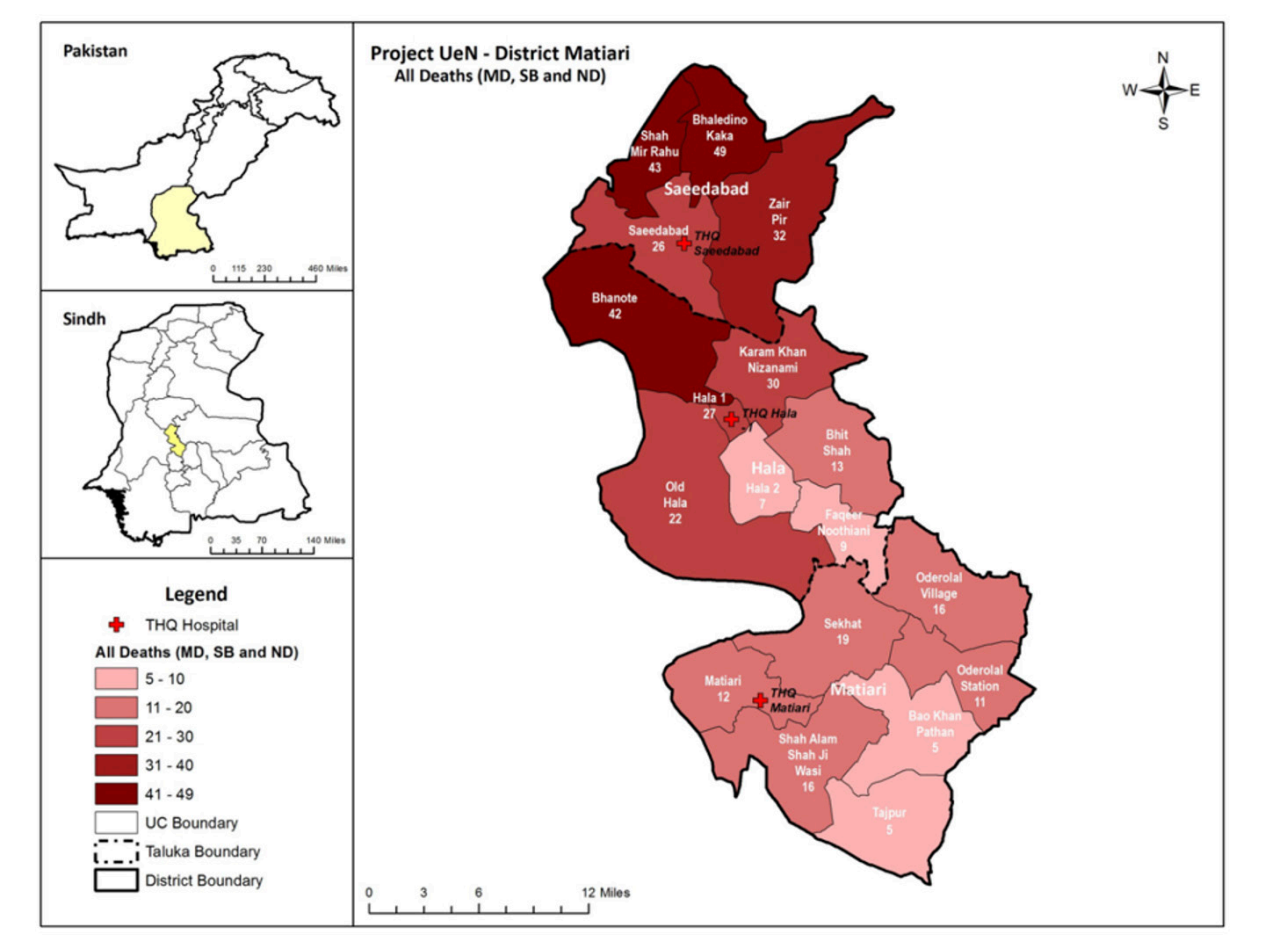
District Matiari, location of THQs hospital, and distribution of death cases among three Taluka.

The audit committee notified 389 deaths in Matiari District, comprising 21 MDs, 140 SBs, and 228 NDs (**Figure 2**). Of these 389 notified deaths, 319 were included in the final analysis.

### Health services indicators

Among 389 cases, 64 (16%) mothers opted for ANC at the health facilities that were the implementation sites of our study. Moreover, 148 (38%) of mothers sought ANC at other government facilities, which were either BHUs (107 cases), RHCs (18 cases), or hospitals (23 cases). Finally, 131 (34%) mothers sought ANC at private health facilities 131(34%), which were either private hospitals (126 cases) or private clinic near home (5 cases). Of 389 notified deaths, 31 (7.9%) occurred at study facilities (implementation sites),, as validated by Matiari’s district health information system. Additionally, 151 (38.8%) deaths occurred at other government facilities, 122 (31.3%) at private health facilities, 11 (2.8%) on the way to hospital, and 53 (13.6%) at home. Of the 389 deaths, 271 (70%) occurred from 2 pm to 9 am. More than two referrals were observed in 290 (74.5%) death cases, *i.e.* 7 MDs, 96 SBs, and 187 NDs (**Table 1**).

**Table 1:** Place of ANC, place of delivery and death, time of death, and number of referrals (n = 389), n (%)

	Overall (n = 389)	Maternal deaths (n = 21)	Stillbirth (n = 140)	Neonatal deaths (n = 228)
**Place of ANC**				
Study facilities	64 (16)	2 (3.1)	31 (48.4)	31 (48.4)
*THQ Matiari*	16 (25)	2 (11.7)	8 (47.0)	7 (41.1)
*THQ Saeedabad*	16 (25)	0 (0)	9 (56.2)	7 (43.7)
*THQ Hala*	31(48)	0 (0)	14 (45.1)	17 (54.8)
Other government facilities	148 (38)	8(5.4)	50 (33.7)	90(60.8)
*BHU*	107 (72)	6 (5.6)	41 (38.3)	60 (56.0)
*RHC*	18 (12)	0 (0)	3(16.6)	15 (83.3)
Government hospitals*	23 (15)	2 (9.0)	6 (27.2)	15 (64)
Private health facility	131 (34)	9 (6.8)	41 (31.3)	81 (61.8)
*Private hospital*	126 (96)	7(5.5)	40 (31.7)	79(62.7)
*Private clinic near home*	5 (3)	2(40)	1 (20)	2 (40)
*Missing data*	46 (12)	2	18	26
**Place of delivery**				
Study facilities	34 (8.7)	0 (0)	18 (52.9)	16 (47.0)
*THQ Matiari*	5 (14)	0 (0)	4 (80)	1 (20)
*THQ Saeedabad*	10 (29)	0 (0)	6 (60)	4 (40)
*THQ Hala*	19 (55)	0 (0)	8 (42.1)	11 (57.8)
Other government	137(35)	9 (6.5)	49 (35.7)	79 (57.6)
*BHU*	44 (32)	2 (4.5)	19 (43.1)	23 (52.2)
*RHC*	15 (10)	0 (0)	2 (13.3)	13 (86.6)
*Government hospitals*	79 (57)	8 (10)	28 (36)	43 (55.2)
Private health facility	143 (36)	6 (4.2)	48 (33.5)	89 (62.2)
*On the way*	9 (2.3)	2 (22.2)	3 (33.3)	4 (44.4)
*At home*	34 (8.7)	1 (2.9)	13 (38.2)	20 (58.8)
*Missing data*	32 (8.1)	2	9	20
Place of death				
Study facilities	31(7.9)	2 (3.2)	19(61.2)	11 (35.4)
*THQ Matiari*	6 (19.3)	1 (16.6)	4 (66.6)	1 (16.6)
*THQ Saeedabad*	8 (25.8)	0 (0)	5 (62.5)	3 (37.5)
*THQ Hala*	18 (58)	1 (0)	10 (58.8)	7 (41.1)
Other government*	151 (38.8)	13 (8.8)	52 (34.4)	86 (56.9)
*BHU*	32 (21.1)	2 (6.2)	19 (59.3)	11 (34.3)
*RHC*	8 (5.2)	0 (0)	3 (37.5)	5 (62.5)
*Government hospitals*	111 (73.5)	11 (10.4)	30 (27.0)	70 (63.0)
*Private health facility*†	122(31.3)	3 (3.2)	48 (39.0)	71 (57.7)
*On the way*†	11 (2.8)	2 (10)	1 (10)	8 (80)
*At home*†	53 (13.6)	1 (1.8)	12 (22.6)	40 (75.4)
*Missing data*	21 (5.3)	0	8	12
Time of death				
*9 am to 2 pm*	91	4 (4.40)	38 (41.7)	49 (53.8)
*After 2 pm to 9 am*	270	15 (5.1)	92 (34.07)	164 (60.7)
*Missing data*	28	2	10	15
Number of referrals				
*≤2*	29	14 (6.6)	11 (37.9)	4 (1.8)
*>2*	290	7 (33.3)	96 (33.1)	187 (82.0)
*Missing data*	70	0	33	37

BHU – basic health unit, RHC – rural health centre, THQ – Taluka Headquarter

*Tertiary level care hospitals and THQs in the nearby cities.

†Based on self-reported information by the respondents.

### Characteristics of reviewed cases and their distribution per the FDM

The audit committee and project analysis teams reviewed and analysed a total of 319 (82%) of the 389 notified deaths after excluding refusals (non-consent), non-respondents (respondent not available even after three visits), and incomplete cases (missing some data). The final sample comprised 19 MDs, 115 SBs, and 185 NDs (**Figure 1**, **Table 2**). No delays were identified in 2 out of 19 MDs, and in 39 of 185 NDs.

**Table 2:** Sociodemographic characteristics of cases reviewed (n = 319)

	Overall (n = 319)	Maternal death (n = 19)	Stillbirth (n = 115)	Neonatal deaths (n = 185)
**Number of pregnancies since marriage (gravidity), x̄ (SD)**	3.41 (2.96)	3.13 (2.36)	3.77 (3.27)	3.15 (2.66)
**Number of living children (parity), x̄ (SD)**	2.51 (2.12)	2.69 (2.02)	2.70 (2.26)	2.36 (2.05)
**Age at first pregnancy in years, x̄ (SD)**	20.68 (4.73)	21.00 (4.72)	21.42 (3.46)	20.22 (5.33)
**How much of the gap was in between previous pregnancy and current conception? (interpregnancy interval in years), x̄ (SD)**	4.14 (1.54)	2.78 (1.79)	3.54 (1.37)	4.49 (1.54)
**Age of respondents, x̄ (SD)**	30.34 (4.61)	28.21 (6.47)	36.46 (4.60)	26.68 (6.46)
**Age group of the respondents in years, n (%)**				
≤20	61 (19.1)	3 (15.8)	19 (16.5)	39 (21.1)
21–30	175 (54.7)	11 (57.9)	59 (51.3)	105 (56.8)
31–40	76 (23.8)	4 (21.1)	35 (30.4)	37 (20.0)
>40	8 (2.5)	1 (5.3)	2 (1.7)	4 (2.2)
**What is the highest level of school attended, n (%)**				
None/preschool	200 (62.5)	9 (47.4)	72 (62.6)	119 (64.3)
Primary	72 (22.5)	7 (36.8)	29 (25.2)	35 (18.9)
Secondary	17 (5.3)	1 (5.3)	4 (3.5)	12 (6.5)
Higher	30 (9.4)	2 (10.5)	10 (8.7)	18 (9.7)

Eight (42%) MDs, 74 (64%) SBs, and 89 (48%) NDs encountered delays in responding to early warning signs. Five MDs (26%) 69 (60%) SBs, and 65 (35%) of NDs saw delays in receiving medical care. Fourteen (74%) MDs, 69 (60%) SBs, and 65 (35%) NDs experienced delays in reaching an appropriate medical facility, with one neonate passing away during the transit to the other healthcare facility. Lastly, 14 (74%) MDs, 40 (35%) SBs, and 100 (54%) NDs experienced a delay in receiving treatment upon arrival at the healthcare facility.

**Figure 3:**
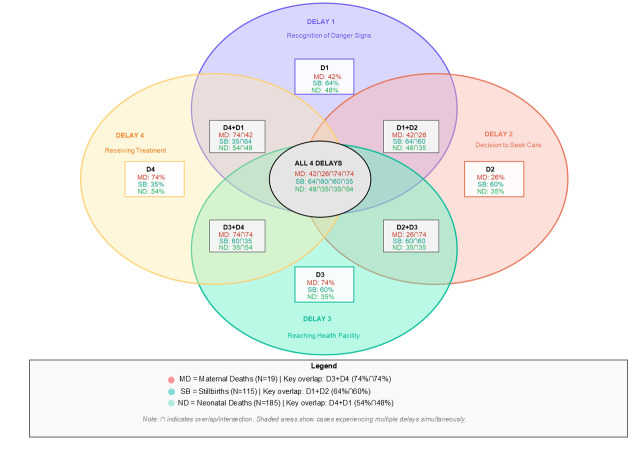
Venn diagram showing the distribution of the four delays (n = 319).

The distribution of delays reveal distinct patterns across all death types. For MD, delay 3 (74%) and 4 (74%) were most prominent, reflecting challenges in accessing facilities and receiving adequate care. The SBs predominantly saw issues with delay 1 (64%) and 2 (60%), indicating failures in recognising complications and deciding to seek care. For NDs, delays 4 (54%) and 1 (48%) were mostly identified. The results showed that 249 of 319 cases (78%) experienced multiple concurrent delays, demonstrating the interconnected nature of barriers across the care continuum (**Figure 3**). The overlap between delays 3 and 4 was particularly common 112 cases (35%).

### Triangulation of quantitative and qualitative findings

Quantitative analysis showed that 271 if 389 deaths (70%) occurred between 2 pm and 9 am, a period that overlaps with times when government facilities typically operate with limited staffing (most facilities function primarily between 8 am and 2 pm). However, this temporal association alone does not establish plausibility: deaths may cluster after hours due to delayed care-seeking, referral delays following daytime complications, or systematically higher severity among evening or night-time presentations.

To strengthen our interpretation, we triangulated findings with qualitative narratives to examine whether facility availability was experienced as a barrier during emergency care-seeking. The qualitative data provided clear convergent evidence. Families frequently described arriving at government facilities during evenings or nights and encountering locked gates, absent staff, or being instructed to return the following morning. Representative narratives included: ‘They didn’t admit her because there was no electricity and no female staff’ (MD-213-0012) and ‘No staff was present as it was Sunday’ (SB-311-0025). While confounding remains possible and definitive attribution would require more rigorous study designs, the convergence between quantitative temporal patterns and qualitative accounts of facility closure provides stronger, though still not causal support for extended facility hours as an intervention priority. Importantly, the integration of both data sources indicates that facility closure was not merely temporally associated with the deaths, but was explicitly encountered by families as a barrier to emergency care.

Multiple referrals between facilities emerged as a second critical barrier, with strong convergence across data sources. Quantitatively, 290 of 389 (75%) cases involved more than two referrals before appropriate care was reached or death occurred. This high prevalence was independently corroborated in the qualitative narratives, where families consistently described fragmented referral pathways involving three to five facility visits. The narratives elucidated the mechanisms underlying this pattern: repeated encounters with statements such as ‘we cannot manage this case’, unclear or absent guidance regarding where to seek care next, lack of coordination or transport support between facilities, and escalating financial burden with each unsuccessful visit. One representative account illustrates this process:

*We took her to three different facilities before finding one that would admit her. At each place they said, ‘go to a bigger hospital’, but didn’t tell us which one or how to get there.* – MD313-0021.

The data triangulation demonstrated that both data sources independently identified multiple referrals as widespread and harmful, with no contradictory evidence. The qualitative data extended the quantitative findings by clarifying not only the prevalence of referrals (the ‘what’), but also the potential causes (capacity limitations at lower-level facilities) and their consequences (delays, costs, low capacity of decision making, and clinical deterioration during transport). This convergence across methodologies provides evidence that fragmented referral systems represent a critical and actionable intervention point (**Table 3**).

**Table 3:** Identifying factors impacting maternal and perinatal deaths, categorised according to the FDM across three levels: individual, community, and health facility

Delay	Individual level	Community level	Health facility level	Representative quote	Participant
Delay 1: recognition of danger signs	Unawareness of danger signs, failure to recognize symptoms, unaware of severity, reliance on home remedies			*She wanted to avoid surgery… drinking milk with ghee… escalated her BP. She always said it’s my normal BP.*	MD- 313-0017, respondent: sister
				*I went to the doctor for a check-up, and the doctor told me that my child died 15 d ago in the womb.*	SB-311-0038, respondent: mother
				*If I had given ORS, my child might be alive.*	ND-312-0054, respondent: mother
Delay 2: decision to seek care	Ignorance/neglect personally, non-compliance with doctor’s advice, fear of mistreatment, lack of caretaker for children/home, lack of companion, misconceptions/beliefs, poverty,	Reluctance due to cultural constraints, lack of encouragement from relatives/community, customs and traditional practices		*She seemed under black magic, so I didn’t take her to the doctor.*	MD-113-0016, respondent: mother of deceased
				*If I had arrived 5 min earlier, the child might have been alive, nobody was at home to take me to the facility.*	SB-111-0040, respondent: mother
				*After leaving for the XXX private hospital at 7 pm, the baby was born healthy at 9 pm, at home… My kid passed away at 10:10 pm*	ND-112-0038, respondent: mother
Delay 3: reaching health facility	Time taken to arrange transport, distance to facility for second referral, cost of transport, personal preference for multiple referrals	Lack of emergency transportation, lack of awareness of existing services, lack of community support		*We took her to ABC private hospital… then to a government hospital in Near City then to XXX private hospital and then to YYY private hospital… finally came to AAA government hospital in Big City… her body was swollen.*	MD-113-0005; respondent: mother-in-law
				*I delivered the dead baby in the car.*	SB-311-0044, respondent: mother
				*My husband went to fetch the car, and I was about to take the child to the hospital, but the child died at home. I am very upset about this loss.*	ND-312-0014; respondent: mother
Delay 4: receiving adequate care at facility			Facility closed, absence of staff, shortage of medicines/blood, lack of emergency equipment, mismanagement in providing care, high costs. delays in admission, no female provider	*They didn’t admit her because there were no electricity and female staff.*	MD-213-0012, respondent: sister-in-law
				*No staff were present as it was Sunday.*	SB-311-0025, respondent: mother
				*They injected medicines to my child, but doctor said they don’t have machinery.*	ND-112-0040, respondent: mother

## DISCUSSION

We implemented the WHO’s death audit review system (audit cycle) [9] at secondary-level healthcare facilities in Pakistan, systematically applying the FDM to 319 maternal and perinatal deaths. Our findings reveal distinct delay patterns across death types, where MDs were predominantly influenced by delays 3 (identifying and reaching healthcare facilities) and 4 (receiving adequate care at health facility). reflecting system-level barriers in access and quality of care. The SBs frequently experienced delays 1 (recognising the danger signs) and 2 (deciding to seek care), highlighting individual and household level knowledge and decision-making gaps. Lastly, the ND were primarily affected by delays 4 (receiving timely care at the facility) and 1 (recognising danger signs), indicating both facility capacity constraints and unawareness. Critically, 78% of cases experience multiple concurrent delays, underscoring the interconnected nature of barriers across maternal and newborn care continuum. These findings suggest that many deaths may have been preventable through targeted interventions addressing the four delays.

These findings align with existing literature highlighting delays in adequate knowledge and awareness among women and men about the availability of healthcare facilities (delays 2 and 3), as well as widespread misinformation regarding pregnancy, childbirth, and postnatal care, contributing to the lack of delivery preparedness and increased maternal and perinatal mortality [30–32]. Studies from similar rural settings have demonstrated that women's limited understanding of danger signs (delay 1) and lack of birth preparedness directly impacts their care-seeking behaviours and health outcomes [19,32]. Previous studies have shown that formal maternal education plays a crucial role in influencing the choice of healthcare facilities for deliveries [33].

Another important finding was that the delays 3 and 4 are related to accessing appropriate healthcare due to limited operating hours of government health facilities in Sindh, Pakistan. These facilities typically provide obstetric services only from 8 am to 2 pm on weekdays, with minimal or no staffing during evenings, nights, and weekends. This can pose a major operational barrier, as many emergencies occur outside these hours. This trend is evident in the studies conducted in other districts of Sindh province and in other provinces such as in Punjab, where health service indicators reflect similar challenges [34–36]. We observed that most deaths occurred between 2 pm and 9 am – the period when most government facilities have limited services. Consequently, women frequently opt for private facilities or home deliveries for childbirth, despite utilising government BHUs for ANC. Our qualitative data provides narrative support for the facility hours hypothesis, whereby multiple family respondents specifically described arriving at government facilities during evening or night hours. The lack of availability of services during critical hours intensifies this trend. A study in Punjab documented similar patterns, where the ‘24/7 BHU’ initiative extending facility hours to provide round-the-clock care was associated with increased facility deliveries and reduced home births, providing quasi-experimental evidence that operational hours influence care access [35]. This demonstrates a need for reforms in healthcare accessibility and operational hours across the country.

The quantitative and qualitative findings highlight that multiple referrals across healthcare facilities pose a severe systems challenge. These referrals transfer patients between multiple institutions highlighting systemic inadequacies in managing complicated cases at the initial point of care. Maternal complications and high-risk cases frequently were due to delays in receiving emergency obstetric care. Similarly, neonatal complications were caused by shortages of skilled personnel, essential drugs, and medical equipment at referral service delivery points. This process often results in adverse outcomes, which communities frequently associate with “medical negligence,” reflecting both delays in seeking care and failures within the healthcare system [37,38].

Another key challenge emerging from our study is the inadequacy of robust data systems for accurately tracking maternal and perinatal deaths, particularly for private healthcare facilities where NDs occur. The limited scope of the DHIS, which focuses on public health facilities and excludes private providers, creates a significant gap in understanding the true burden of early NDs and SBs [13,39]. This lack of comprehensive data hinders effective planning and implementation of proven strategies to improve maternal and perinatal health outcomes [4]. There is an urgent need for inclusive data collection systems that integrate private healthcare data to provide a complete picture of health events. Together, these findings indicate that interventions must operate simultaneously across household, transport, and facility levels, rather than targeting individual delays in isolation.

### Strengths and limitations

This study is distinguished by the active and continuous participation of local community representatives, providing empirical support for engaging community members, healthcare providers, and policymakers in identifying preventable factors contributing to maternal and perinatal deaths. Our findings emphasise the necessity for actionable solutions to reduce maternal and child mortality by strengthening health systems, particularly in regions where comprehensive national-level mortality data are lacking. Our findings advance the understanding of maternal and neonatal mortality reduction efforts using a community-engaged and trust building approach. The lessons learned facilitate a comprehensive understanding of mortality factors beyond clinical aspects using narratives from the community respondents [27,32]. The qualitative findings provide deep insights into the underlying factors of maternal and neonatal mortality which are difficult to identify and assess by quantitative research methods alone and often can undermine the success of clinical interventions [40]. These factors support the utility of the FDM and provide evidence for critical intervention points to reduce maternal and infant mortality. Furthermore, the study’s focus on secondary-level facilities in a rural district generates valuable evidence for similar resource-constrained settings. Our results show that implementation of context-specific solutions through local audit committee representatives can reinforce the community engagement and inclusion of respected local figures enhances trust and early referral decisions.

This study has several strengths. We enhanced methodological rigor through standardised translation procedures from Sindhi to English by using a standardised codebook, meeting frequently, sharing and comparing the results. Throughout the study, we conducted an ongoing internal quality audit, adapted from O'Cathain *et al*. [25,41] to determine whether the data were collected, analysed, and reported correctly according to the study protocol [24]. The team discussed our researcher’s positionality as an acknowledgment that the researcher's identities, experiences, beliefs, and social location (like gender, race, class, culture) inherently can shape their research process, influencing how they interpret findings, moving beyond claims of pure objectivity to embrace reflexivity and transparency in qualitative research [42].

This study also has several limitations. First, the project team was actively notified about each death case by the community representatives and cross referenced validate these reports by examining the health facility registers. This approach may have missed cases due to potential underreporting caused by fear and lack of robust data from private facilities. The deliveries reported are from the catchment population of the three THQs and LHWs linked to these facilities. However, the use of the LHW’s management information system as the delivery denominator introduces selection bias, as it primarily captures deliveries from areas covered by LHWs. Although the project team actively notified cases, this approach may still lead to an overestimation of the true mortality rates. Second, the case tracing and retrieving health services indicators presents challenges, with some missing, incomplete, or unclear records, particularly in terms of clinical diagnosis. Additionally, we were unable to ascertain the mortality rate due to uncertainties surrounding the denominator. Instead, our analysis focuses on characterising the patterns of delays among notified and reviewed deaths and identifying modifiable factors contributing to mortality that do not require precise rate calculation. Third, a substantial portion of the contributing factors derived from the qualitative narrative relies on participants' perceptions, introducing the possibility of perception bias [43]. Fourth, conducting interviews after the harmful events raises concerns about recall and trauma bias. We tried to address this challenge by adding an additional person from the family during the interviews to avoid the possibility of incomplete responses [44]. Fifth, we did not directly assess the actual treatment quality received at health facilities, but relied instead on the reported information as provided by the patients or family member respondents. Sixth, the absence of data pertaining to women who survived their delivery poses a limitation, potentially compromising the full understanding of the prevalence of risk factors associated with maternal emergencies. Seventh, we adopted a deductive approach, which led participant narratives being primarily assessed for alignment with pre-established themes related to the four identified delays and making them prone to surveillance bias. Nevertheless, our findings are robust due to our integrated mixed-methods approach, the validation of our research team, and expert review, and inputs from the community. They are also relevant to other rural areas in Pakistan and other LMICs due to the shared socioeconomic and cultural factors [45]. Eighth, the quantitative analysis was limited to descriptive statistics and exploratory comparisons due to small sample sizes, particularly for MDs (n = 19). This sample resulted in wide confidence intervals (typically ±20–25 percentage points for maternal death delay prevalence estimates), limiting our statistical power to detect differences between maternal deaths and other death types. Observed differences in delay patterns should, therefore, be interpreted as exploratory and hypothesis-generating rather than definitive. Subgroup analyses would be underpowered even for SBs (n = 115) and NDs (n = 185). We limited our analysis to overall delay patterns, rather than examining delay distributions within demographic or clinical subgroups. Ninth, we saw substantial missing data for health service indicators (7–22% across variables), reflecting systemic documentation gaps in rural Pakistan's health system, rather than data collection failures. We addressed this through verbal autopsy narratives that followed the WHO methodology [27,28] which prioritises family accounts of care pathways over specific clinical data points. However, families’ inability to recall precise referral sequences or exact timing may have resulted in the underestimation of delay severity, particularly for complex multi-facility pathways. Finally, the impact measurements in terms of maternal and perinatal death rates were not directly ascertained due to the short duration of the study. However, the high burden of delays suggests substantial room for improvement.

## CONCLUSIONS

Our findings demonstrate that the FDM could identify distinct patterns of preventable factors contributing to maternal and perinatal deaths across household and health system levels in Pakistan. Based on these findings, audit committees generated actionable recommendations, demonstrating potential for systematic learning from deaths in resource-constrained settings (**Box 2**). Health systems need to improve and invest in women’s access and availability of healthcare facilities both during and after pregnancy. Embedding audit systems within routine district health governance could enable continuous learning and targeted interventions in similar resource-constrained settings.

Box 2 **Addressing the factors that contribute to maternal and perinatal deaths**

A distinctive aspect of our study is the pragmatic solutions proposed by the local audit committee members to address the factors they documented for the delays in accessing maternal and neonatal care in District Matiari, Sindh, under the supervision of a district health officer. These solutions targeted the four delays directly and highlight the critical roles of community engagement and education, comprehensive maternal healthcare services, and the importance of timely medical referrals as preventive measures against maternal and neonatal mortality. The local leaders emphasise on raising awareness about the warning signs of stillbirth and the necessity to provide high-quality care to neonates at health facilities was particularly notable. Participants were motivated to work collectively to generate and document specific actions to mitigate the critical delays and avoidable delay factors as they saw them.

Women and men actively participated in awareness sessions conducted by lady health workers and physician members of audit committee to address delay 1 (recognition of danger signs). Overall, 45 sessions were conducted (three sessions per month) which focused on the importance of recognising danger signs during pregnancy and for newborns, early decision-making in referrals, transport arrangements near the expected date of delivery, newborn care, hygiene and nutrition, and counselling for family planning and avoidance of early marriages. The deliberate selection of active community members helped to build trust, confidence, a strong link with the facility, and reinforced importance of community-led efforts to address delay 2 (decision in seek care). An ambulance service, connected through a community representative from the audit committee, was established at one of the project’s secondary-level hospitals to address delay 3 (reaching health facility). Administrative steps were taken at the facility level, including the timely provision of referral slips, antenatal cards, district health information system forms at the outpatient departments and wards, solar panel installation, landline phone activation, and intra/inter-facility communication to avoid delay 4 (receiving care at facility). Recommendations proposed to the district health office by the audit committee included training of sonographers, implementing a rotation system for medical officers during evening and night shifts, and recording detailed medical histories to improve diagnostic accuracy, ensure continuity of care, and enhance health outcomes.

## Additional material


Online Supplementary Document
