# Supplementary material for: Identifying delays impacting maternal and perinatal deaths using a facility-based death audit review system integrated with community engagement: a mixed methods study
Source: J Glob Health. 2026 Apr 24;16:04117. doi: 10.7189/jogh.16.04117 (PMC13108537; doi:10.7189/jogh.16.04117)
Supplement: Online Supplementary Document [file jogh-16-04117-s001.pdf]

Supplement to: Cite as: Memon Z, Ahmed W, Muhammad S, Soofi S, Chouhan S, Rizvi A, Barach P, Bhutta Z. Identifying delays impacting maternal and perinatal deaths using a facility-based death audit review system integrated with community engagement: a mixed methods study. J Glob Health. 2026;16:04117.

Good Reporting of a Mixed Methods Study (GRAMMS) checklist

**Guideline**	**Section: page**
Describe the justification for using a mixed methods approach to the research question	Methods<br>- pg. 4 &16
Describe the design in terms of the purpose, priority and sequence of methods	Methods- pg. 4 - 6 , Figure 1
Describe each method in terms of sampling, data collection and analysis	Methods-Informed consent and data collection procedures: pg 7-9<br>Quantitative and Qualitative Analysis: pg. 10-11
Describe where integration has occurred, how it has occurred and who has participated in it	Methods- pg. 12-14, Table 3
Describe any limitation of one method associated with the present of the other method	Discussion pg. 16
Describe any insights gained from mixing or integrating methods	Discussion: pg. 15-17

O'Cathain A, Murphy E, Nicholl J. The quality of mixed methods studies in health services research. J Health Serv Res Policy. 2008;13: 92-98.

Study Variables and Definitions S1 Online Supplementary Document

Identifying delays impacting maternal and perinatal deaths using a facility-based death audit review system integrated with community engagement: A mixed methods study

Study Variables and Definitions

Variable	Definition
**Sources of Notification**	The death cases were notified by death community representatives from community, Lady Health Worker Management Information System (LHW-MIS), Health care providers from three secondary level care health facilities and records in District health Information System (DHIS).
**Project Research team**	Project research team comprise of two district field managers, one quality assurance officer, one research associate, five trained data collectors, one research manager, one senior manager and one operational director and the project supervised by Principal Investigator.
**LHW**	Lady Health Worker is the community health worker, expected to record all births and deaths in her allotted catchment population. Usually covers 100-150 households. Also register eligible couples, counsel couples of reproductive age about family planning methods, keep checks on pregnant women and their nutrition, provide treatment for basic ailments and refer patients to the formal health system, encourage breastfeeding. She reports to lady health supervisor.
**LHS**	Lady Health Supervisor another cadre of community health workers working in the LHW program who are responsible for directly managing 25-30 LHWs. LHSs make monthly visits to each LHW to supervise their community case management (CCM) skills during visits to community households. LHS provide supportive supervision, on job training, verbal and written feedback to her Lady Health Workers.
**LHV**	The LHV will be responsible for providing care under the direct supervision of Medical Officer at healthcare facility by ensuring patients receives adequate services during pregnancy and childbirth, family planning, provision of ANC/PNC services and neonatal resuscitation and postpartum follow up of women
**Implementation Sites (THQ Matiari, THQ Saeedabad, THQ Hala)**	Three tehsil headquarters THQs, secondary level care hospitals in District Matiari were the Death Audit Review System implementation sites. The three facility-based audit committees were established with community representatives from the catchment population. During the project THQ Matiari upgraded to District Headquarter Hospital (DHQ).
**Other Government health facilities**	The other government health facilities include Basic Health Unit (BHU) and Rural Health Center (RHC), Tertiary level care hospitals and THQs in the nearby cities (Hyderabad and Tandoallah Yaar). The information and data collected from communities mentioned these facilities from where they visited for Antenatal Care (ANC), delivery and Postanatal Care (PNC) and seek other maternal and newborn health care services.
**Private Health Facility**	These referred to private hospitals where admission facilities were available. Private clinics referred to as the one room allocated for the health care services provision in the area by one doctor (general physician or gynecologist) with maternity services (ultrasound).
**Death Time**	Death time divided into two categories; 9am to 2pm – this is official timings of the government health facilities.<br>After 2 pm to 9 am – usually LHV and specialists were on-call during this time at the government health facilities.<br>In private hospitals the same pattern followed.

**Referrals**	This refers to the number of times a patient is referred from the first health facility they contacted to the final health facility where they received care. The analysis looks at the proportion of referrals that were less than 2 times versus more than 2 times

Questionnaire S1 Online Supplementarty Document

Identifying delays impacting maternal and perinatal deaths using a facility-based death audit review system integrated with community engagement: A mixed methods study

Stillbirth and Neonatal, Maternal Death Case Review Form

Study ID:_ _ _-_ _ _ _

Case to be reviewed:	Stillbirth........................ 1<br>Neonatal Death ....2<br>Maternal Death 3

STUDY ID KEY	
Taluka	1. Matiari<br>2. Hala<br>3. Saeedabad
Form filled from	1. Community<br>2. Health facility
Case type	1. Still Birth<br>2. Neonatal death<br>3. Maternal Death<br>4. Maternal Near-miss
Case number	Example: 0001, 0002, 0009, 0011 etc.

**Section 1: Household Information**			
Read the consent form (hard copy) to the respondent if 18 -49 years; take consent. Ensure that their name recorded in the consent form if they agree to participate.			
If the woman is 15-17 years old and married, read the consent script to the head of the household/ husband and the assent script to the woman. You MUST obtain the consent of the guardian and assent of the woman before proceeding<br>If the woman is married, or is unmarried and older than 17 years, read her the woman's consent script. You MUST obtain the woman's consent before proceeding<br>May I start now?<br>• Yes, permission is given Begin the interview.<br>• No, permission is not given End the interview.			
**S. No**	**Question**	**Response**	**Skip**
CE101	Survey Number	Pre-Intervention 1<br>Post-Intervention 2	
CE103	Case Notified by	1. LHW<br>2. Relative/friend<br>3. AKU team<br>4. Community Audit members<br>5. Health facility Audit member<br>6. Health facility<br>7. THQ Matiari, THQ Hala ,THQ Seedabad<br>8. Other	
CE104	Village Name رها ڳوٺ جو نالو؟		User input
CE105	Household Number	[_] [_] [_] [_] -[_] [_] [_]<br>Don't know....98	
	Relation with deceased? (specially for Maternal death)		

	گذاري ويل سان ڙ يڪو رشتو آي توهان جو؟		
CE106	Interviewer's ID<br>KK..1<br>YT…2<br>SC…3<br>WA…4	[_]	
CE107	Date of Interview<br>انکويو جا تاريييخ؟	Day [_] [_]-Month [_] [_]-Year [_] [_] [_] [_]	
CE108	Interviewer's Name<br>انکويو وننڊڙ جو نالو؟		
CE109	Interviewer's Phone Number	[_] [_] [_] [_] -[_] [_] [_] [_] [_] [_] [_] [_]	
CE110	Time at beginning of Interview انکويو سوع ڙيتجو وقت؟	HH [_] [_]: MM [_] [_]	
CE111	Time at end of Interview انکويو ختم ڙيتجو وقت؟	HH [_] [_]: MM [_] [_]	
CE112	During this interview was privacy ensured?<br>کیھ اس ان ٹرویو ک دو ان عو ت ایل یھ ؟<br>**چا انکويو وٺڻ دوران عورت ا يي ھھ؟**<br>Observe: was anyone else present and listening during the interview other than the woman being interviewed?<br>مشھہدہ کرے : کیھ اس ان ٹرویو ک دو ان کوی او شخص موجود یھھ او<br>سن ہھ یھھ اس عو ت ک علوہ جسکھ ان ٹرویو لیھ جھ ہھ یھھ ؟	Yes 1<br>No 2	IF 1 go to WI101
CE113	Who else was present during the interview?<br>ان ٹرویو ک دو ان او کون موجود یھھ؟<br>**انکويو وٺڻ دوران ڙک ويٺل هو يا هئا؟**<br>Select all that apply<br>یمھم جوابھتکھ انتخھب کریں جو لگو ہو ے ہ یں<br>**جيي لگو ڙت اي سب ٽک يو.**<br><br>Observe: was anyone else present and listening during the interview at any time?<br>مشھہدہکرے : کیھ ان ٹرویو ک دو ان کئ یھ وقتکوی او موجود<br>یھھ /یھ او سن ہھ یھھ /یھ؟	Husband…..1<br>Children……2<br>Other Male Relative…..3<br>Other Female Relative….4<br>Other (Specify)….96	
CE114	Result of Woman's Interview<br>**انکويو جو چا نتيجو نکتو؟**	Completed 1پو وتیو<br>Not at home 2گھر کھن رھ<br>Not competent 3انرویو ڈیڻ کھبل ن رھ<br>Deferred 4ٿھ ي چڏیو<br>Refused 5انکھ کري چڏیو<br>Partially completed 6 اڌپو و ریو<br>Other (specify) 96	

Section 2: Woman's Information Module			
**S. No**	**Question**	**Response**	**Skip**
CE201	In what month and year were you born?<br>**توهان پيدائش تاريييخ چا آي؟**<br>آپ ک پیداشکس مھیے او سھل م یں ہوی یھ؟<br>If day is not known type 98, if month is not known type 98, if year is not known type 9998<br>اگر خر نھي ت 98 لکو.	Day [_] [_]-Month [_] [_]-Year [_] [_] [_] [_]	
CE202	How old are you?<br>**توهان ععمگگ يھ آي؟**<br>آپ ک عمرکتھ ے ؟<br>Probe: how old were you at your last birthday?<br>پوچھ یں: آپ ک آخری سھلگر ہ پر عمرکتھ یھ ؟	Age (in Completed Years)	
CE203	Have you ever attended formal education?<br>**توهان ي تکا پڙهيل آهيو؟**<br>کیھ آپ ے کیھ سم یعلیم حھصل ک ے ؟	Yes ہھں 01<br>No نہ یں. 02	(if 2 skip to CE205)

CE204	What is the highest level of school you attended?<br>يهڙي لس تا ڙت؟<br>آپ ے اسکول ککس لیول یک پڑرھ؟	Preschool پری اسکول 01<br>Primary پرائمری 02<br>Secondary سیکنڈ ی 03<br>Higher اس س زیھده 04<br>Refuse to answer جواب دیے س انکھ کیھ 88	
CE205	What is your current marital status?<br>هن وقت توهان اادوا حيصيت چا آي؟<br>اس وقت آپ ک ازوای حیثیتکیھ ے ؟	Currently married اس وقت شهدی شده 01<br>Widowed بیوه 02<br>Divorced الق یهفته 03<br>Separated شوہر س الگ ہ تھ ے 04<br>Refuse to answer جواب دیے س انکھ کیھ 88<br>Other (specify) دیگر وضهحت کریں 96	Only for 01 CE 206 will be asked
CE206a	What was your age at the time of first marriage?<br>شادي ڙ يٺ وقت توهان عمر ي ڪي هي؟(يا شاديڪگتا سال (يا آهن؟<br>شهدی ک وقت آپ ک عمرکیھ یھ؟	Day [_] [_]-Month [_] [_]-Year [_] [_] [_] [_]<br>**Or**<br>Age (in Completed Years)	
CE 206	Are you or your husband currently doing something or using any method to delay or avoid getting pregnant? (before the last pregnancy)<br>چاتوهان اال مڙس ا حعل ۾ وقق (فيعي النڱ) جو طريقو استعمال مي رهيا آهيو؟اگم ها ئ يهڙو طريقو؟ کیھ اپ یھ آپ ک شوہری الحهل حمل م یں وقے ک ل ےکوی اریقه استعمهلکر ہ ہ یں؟	Yes ہهں 01<br>No نه یں. 02<br>________________________________	
CE207	Does any member of your household own: ?<br>چا توهان گگم ۾ نهن فمد وٽ اي شيون آهن؟<br>کیھ آپ کگهر ک ئ فرد ک پهس یه چ یریں اپھ ہ یں؟	Watch گھڑی (Yes/No)<br>Bicycle بهئیسکل (Yes/No)<br>Motorcycle / Scooter موٹر سهئیکل یھ اسکوٹر (Yes/No)<br>Car / Truck/ Jeep گھڑی ،ٹرک یھ جیپ (Yes/No)<br>Animal-drawn cart جهنو وں س چلی جهے وال گھڑی (Yes/No)	
CE208	Who usually makes decisions about making major household purchases?<br>گگم ۾ نهن وڏي خريداري بابت ڙڪ فيصلو ندو آي؟<br>گھر م یں یڑی خریدا ی ک متعلق فیےل عهم او پرکون کریھ ے ؟<br>Select one<br>ایک جوابکھ انتخهب کریں<br>ا ٧ هڪ جواٻ چونڊ ريو .	Respondent جواب دہنده 01<br>Husband شوہر 02<br>Respondent and Husband جواب دہنده او شوہر 03<br>Father or Father-in-law والد یھ سس 04<br>Mother or Mother in-law والده یھ سهس 05<br>Other male family member فیمل ک دوسے مرد 06<br>Other (specify) دیگر وضهحت کریں 96	
CE209	Who usually makes decisions about health care for yourself?<br>توهان صحت باري ۾ سار سنڀال حواي سان ڙڪ فيصلو ندو آي؟<br>آپ ک صحت او دیکھ یهھل ک متعلق فیےل عهم او پرکون کریھ ے ؟<br>Select one<br>.ڙڪ جواب ی چونڊ ڪريو	Respondent جواب دہنده جواب ڏيندڙ01<br>Husband شوہر مڙس02<br>Respondent and Husband جواب دہنده او شوہر جواب ڏيندڙ ۽ مڙس03<br>Father or Father-in-law والد یھ سس 04<br>Mother or Mother in-law والده یھ سهس 05<br>Other male family member فیمل ک دوسے مرد (خهندان جھ ٻیھ مرد)06<br>Other (specify) دیگر وضهحت کریں 96	
CE210	Wi6. Who usually makes decisions about health care for your children?<br>توهان ٻارن صحت حواي سان ڙڪ فيصلو ندو آي؟<br>آپ ک بچوں ک صحت او دیکھ یهھل ک متعلق فیےل عهم او پر کون کریھ ے ؟	Respondent جواب دہنده 01<br>Husband شوہر 02<br>Respondent and Husband جواب دہنده او شوہر 03<br>Father or Father-in-law والد یھ سس 04<br>Mother or Mother in-law والده یھ سهس 05<br>Other male family member فیمل ک دوسے مرد 06<br>Not applicable قهبل اا لق نه یں...65	

		Prefer not to answer جواب دینھ پسند نہ ہی<br>کرونی ....66<br>Other (specify) دیگر وضھحت کریں 96	
CE211	How far do you live from the closest health facility?<br>توهان گگم سان صحت جو مم ز ي تکو پمي آي؟<br>آپ گگھر س سب س قريهھ مرکز صحت کے فھصل پر ے؟	<1.0km ايککلو مي ٹر س کم 01<br>1.0-2.5km ايک س ڈرھيکلو م ی ٹر ک د ميهن 02<br>2.5-5.0km 2.5 کلو م ی ٹر س 5 کلو م ی ٹر ک د ميهن 03<br>05 5.0-7.5km 5 کلو م ی ٹر س 7.5 کلو م ی ٹر ک د ميهن 04<br>>7.5km 7.5 کلو م ی ٹر س زيهده فهصل پر<br>Don't know معلوم نہ ہی 98	
CE212	Which health facility you prefer and visit for ANC?<br>يورهن کيهڙي صحت ی مرکز تی ذيکهہ يندا آريو؟	Nearby Government facility گھر ی پر وا ي<br>سکھ ي اسپتھل---------------- ی---01<br>Nearby private facility گھر ی ء وا ي خھنی<br>اسپتھل--------------------------02<br>Other facility GF or PF far away from own city/town/ ی شہر وا ي سکھ ي يھ خھی اسپتھل -----<br>-------------------------------03	
CE213	Reason for preferring selected (in CE212)facility?<br>توهان شهم (پمي) اسپتالي جو تمجيع ڏيو ا؟	1- Medicine available دوا مل ی<br>2- Doctor/nurse/LHV available at any time صحت کھ کن موجود روندا آرن<br>3- Doctor/nurse/LHV positive response صحت کھ کن جو ويو سنو روندو آي.<br>4- Person preferences موںی ارھ مرکز و ٹی ی<br>5- My husband/family prefers مڑس /گھر وا ن ک ارھ مرکز و ٹی ی<br>6- previous bad experience in nearby facility نذديک ی مرکز م پراٹو يجربوخراب ريو<br>7- Other(specify) --	

Pre-Birth (Antenatal) and Childbirth Module ماڈل ماٹ چکاس حعل دوران صحت			
**S. No**	**Question**	**Response**	**Skip**
CE301	Survey Number	Pre-Intervention 1<br>Post-Intervention 2	
CE302	Facility Name		
CE303	Facility ID	[_] [_] [_] [_] [_] [_]	
CE304	Village Name		
CE305	Household Number	[_] [_] [_] [_] -[_] [_] [_]	
CE306	Woman's ID: عو ت ک آی ڈی		
CE 307	How many times have you been pregnant since your marriage? (INCLUDING CURRENT)<br>توهاڻ پيهڻ شادي کان پو ۽ ي تکي دفعا حعل يو آي؟<br>آپ اپھ شھدی ک بعد س کتھ بھ حھمله ہوی ہ يں؟<br>How many children are alive now? (Parity)<br>ي تکا بار هينک اندا اهن؟	[_] [_] [_] G<br><br>[_] [_] [_] P	
CE308	What was your Age at first pregnancy?<br>پيهرين حعل ز يٺ وقت توهان ععمگگ يھ هھ؟	Years----------months--------	
CE 308 a1	How much gap was in between previous pregnancy and current conception?<br>پوا ڙت حعل ۽ هن حعل وچ ۾ ي تکو وقفو هئو؟	Years----------months--------	
CE308	What was the month and year of your most recent	Date of last delivery جھليه ڈيليو ی ک يھ ييبيخ	

a	delivery?<br>بھ کيھڙي يھ يييبخ تی)آخمی ويم جو مھينو، تاريييبخ ۽ وقت ٻڌايو؟<br>جھئو)<br>آپ ک حھليه ڈيليو ی ک يھ يييبخ او مہينہکونسھ يھھ ؟<br>I mean the last time you gave birth, even if the child is no longer living, or whose father is not your current husband<br>م يرا مطلب آپ ک آخری ڈيليو ی س ے اگرچه اب وه بچه زنده نه يں يھ اسےکھ والد آپکھ شوہر نه يں<br>**If respondent does not know exact date, probe to estimate month and year**<br>اگر جواب دہنده کو صحيح يھ يييبخ معلوم نه يں يو مہينه او سھلکھ اندازه لگھے ک ل ے مزيد يےيل پوچھں	Month مہينه _____<br><br>Year سھل __________	
	Time of delivery:	…...................... am/pm	
CE309	Is your last-born child (Name) still alive?<br>**چا توهان جو آخمي پيدا يل ہار ها ي انداھ آي؟**<br>کيھ آپکھ آخری پيدا ہو ے وا ل بچه /بی (نهم) ايھ يھ زنده ے ؟	Yes, ہہھں 01<br>No نه يں 02	If 1 go to CE310a
CE 309a	Gender of baby	Male…1<br>Female…2	
CE309b	What was the weight of baby at time of birth?	Less than 2.5grams<br>More than 2.5 grams<br>Don't know….98	
CE310	How old was (Name) when she/he died?<br>**ممڻ وقت ہار عمم چا هھ؟**<br>مر ے وقت اس بچه /بی ک (نهم) عمرکيھ يھ؟	Age (days, hours, minutes) ________________<br>Immediately after birth پيداش ک فو ا بعد ……………01<br>Within 7 days of birth پيداش ک 7 دن اند ……………02<br>Within 28 days of birth پيداش ک 28 دن اند ………..03<br>Born dead (Still birth)………04<br>(مرده پيدا ہوا يھھ (مرده پيداش(<br>After 28 days of birth پيداش ک 28 دن بعد) 96<br>Other specify__________________	
CE310a 1	Where (name) died?<br>**نالو) يھ ۽ يھڙي ٽاايم گذاري ويو؟)**	**Home گھم**<br>Your home اپےگھر پر 01<br>Other home کئ او کگھر پر 02<br>**Public sector عوام شعبه**<br>Government hospital (THQ Matiari, Hala and Saeedabad) سکھ ی ہسپتھل 03<br>05 RHC/ MCH آ ايچ س /ايم س ايچ 04<br>ی ايچ يو/ايف ڈبلو س BHU/FWC<br>Other public (specify) (ديگر سکھ ی (وضھحت کريں<br>06<br>AKHSP facility اے ک ايچ ايس يکھ مرکز صحت 07<br>Private hospital/clinic نی ہسپتھل /کلينک 08<br>CMW clinic کيمون ٹھ مڈوائفکلينک 09<br>Other private (specify) (ديگر نی (وضھحت کريں<br>10<br>Hakeem حکيم 11<br>Other (specify) (ديگر (وضھحت کريں 96	
CE310b	At what time ?	Time and date of death----------------------------	
CE310a	What was the gestational age of (NAME) at birth?<br>**حعل مدت چا هھ(نالو) پيدائش وقت؟**<br>نهم) ک پيداش ک وقت آپ ک حمل ک مدتکيھ يھ؟	Day [_] [_]or Month [_] [_]or Weeks [_] [_]	

CE311	While you were pregnant with (name) did you see anyone for pre-delivery (antenatal care)?<br>حعل دوران چا توهان يهـ ڀي پنهنجو طهه معاانو مايو؟<br>جب آپ (نهم) ک سهيھ حهمله يهـ يوكيهـ آپ ے کیٔ س اپنهہ اهـ معهئنه کرایهہ یهہ ؟	Yes ہیهں 01<br>No نہ یں 02<br>Don't know معلوم نہ ہی 98	IF YES GO TO CE313
CE312	Why did you not see anyone for antenatal care during your pregnancy with (name)?<br>هن حعل دوران توهان نهن صحت ار ن سان طهه معاانو چو ى مايو؟<br>نهم) ک سهيهـ حمل ک دو ان آپ ے اپنهہ اهـ معهئنهکیٔ س کیوں) نہ یں کروایهہ ؟	Cost too much ے يہت زيهده قيمت 01<br>Facility not open مرکز صحت کھلنہ ہی 02<br>Too far یہت دو 03<br>Don't trust facility مرکز صحت پر اعتمهد نہ یں 04<br>No female provider at facility مرکز صحت پر صحت ک ديكهہ يهھل ک ل ے عو ت موجود نہ یں 05<br>Husband/family did not allow شوہر /فيمل ے اجهزت نہ یں دی 06<br>Not necessary رو ی نہ ہی 07<br>Not customary واج نہ ہی 08<br>No transportation ٹراسپو ٹ نہ ہی 09<br>Poor quality service صحت ک سهوليهتكهـ خراب معيهـ 10<br>Lack of privacy .... 11<br>ی معلومهت ک حفهظت ک کم/پردےکهـ انتظهم نہ یں<br>Poor attitude of staff استهفكهـ خراب ويه 12<br>Other (specify) (ديگر (وضهحت کریں 96	
CE313	Where did you receive antenatal care for this pregnancy?<br>هن حعل دوران توهان يهـ پنهنجو طهه معاانو مايو؟<br>اس حمل ک دو ان آپ ے اپنهہ اهـ معهئنه كهں کروایهہ ؟<br>Probe for all sources of care, and record all answers given<br>ڏيان سان سب گالهيون پڇو ۽ سب جواب ر ارڊ ريو.<br>ديكهہ يهھل ک يمهم ذ ائع ک متعلق پوچھ یں او يمهم جوابهت يكهہ ڈ کریں	Home گھر<br>Your home اپےگھر پر 01<br>Other home کیٔ او گھر پر 02<br>**Public sector** عوام شعبه<br>Government hospital (THQ Matiari, Hala and Saeedabad) سکهہ ی ہسپتهل 03<br>RHC/ MCH آ ایچ س /ایم س ایچ 04<br>BHU/FWC ی ایچ یو/ایف ڈبلو س 05<br>Other public (specify) (دیگر سکهہ ی (وضهحت کریں-<br>------------------ 06<br>**Private sector** نن شعبه<br>AKHSP facility اے ک ایچ ایس يكهہ مرکز صحت 07<br>Private hospital/clinic ی ہسپتهل /کلینک 08<br>CMW clinic کیمون ٹھ مڈوائفكلينک 09<br>Other private (specify) (ديگر ی (وضهحت کریں --<br>---------------- 10<br>Hakeem حكيم 11<br>Other (specify) (ديگر (وضهحت کریں 96	
CE314	WHOM DID YOU SEE for ANC?<br>نهن توهانجو معاانو يو؟<br>آپ ےکس س معهئنه کروایهہ یهہ ؟	Health Personnel صحت ک اہل کهـ<br>Doctor ڈای 01<br>Nurse/community midwife/LHV 02<br>نرس/کمیون ٹھ مڈوائف/ایل ایچ وی<br>Other person دوسا شخص<br>Traditional birth attendant/ Dai دای 03<br>Lady health worker لیڈی ہیلتهہ و کر 04<br>Hakeem حكيم 05<br>Dispenser/compounder ڈ سپرس /کمپهونڈ 06<br>Relative/friend شته دا /دوست 07<br>Other (specify) دیگر وضهحت کریں 96	
CE 314a	What was the Reason for first ANC visit?<br>پهرين دفعا طهه معاانو ماان چا وجيه هھ؟	To confirm pregnancy حمل ی پک کرنٹ ل ءء.......................01<br>On experiencing of Danger Signs (pain in stomach, severe headache, blurred vision, vaginal bleeding or discharge) صحت ک خطرو محسوس ٹ ی ٹں ی (پیٹ م سو ،مهہ م شدید سو ، انک یں	Multiple responses please 1 only

		ی اگیهن ڏنڊ اچڻ، ٻچيدای مهن خون يھ يھ ٹی جو اخراج<br>02............ (ٹ ی ٹں<br>Due to any known medical reason<br>high blood pressure 03a<br>diabetes 03b<br>kidney problem 03c<br>ڪنهن ی جهٹيل صحت سهن لڳهپيل any other 03d مسئل ی ل<br>ءء(رھ ءء بلڊ پري يس،شگر، گڙدي ی مسئل يھ<br>بيوارڙو<br>(ڪجھ ....<br>To registration for Place of birth ويم ل ءجھ ءمقر<br>(ڪرڻ ل ءء ....04<br>For medicines or supplements اهقت جون دوائون<br>(ءگو يون وٹڻ ل ءء ....05<br>Free labs مفت ٹيسٹون ڪرائڻ<br>ل ءء..06<br>Screening for anomaly in baby ٻھ ۾ ڪنهن نقس<br>ی جهچ ل ءء ....07<br>Friend or relative asked me to<br>visit (دوست/ شتيدا ن ی چوڻ/مشعو ي ٹی .....08<br>To avoid complications during pregnancy and<br>labor حمل ۽ ويم ۾ پيش اچڻ وا ي خطرن سهن بچڻ<br>ل ءء..09<br>I can afford so I visited مهن خرچ برداشت ڪري<br>(سگھهن ی ....10<br>I Know that ANC visits are important for<br>health of mother and baby موني خر آي ۑ حمل<br>دو ان اھ معهئنومهء ۽ ٻھ ی صحت ل ءء لزم<br>آي............11<br>Otherی ڪھ وجی 96	
CE315	How many weeks or months pregnant when you first received antenatal care for this pregnancy?<br>**پهرين دفعا طھ معااھ وقت ي تڪا مهينا يه هفتا حعل هئيو؟**<br>جب آپ ے اس حمل ک دو ان پہل دفعہ معهئنہ کروایھ یو آپکھ حمل کۓ ہفتوں یھ مہے کھ یھھ؟<br>Record the answer as stated by respondent.<br>مزید پوچھ یں : جواب یکھ ڈکریں جس جواب دہندہ ے یتھیھ ے۔	Weeks ہفتے<br><br>Mont hs مہیے<br><br>DK معلوم نہ یں 98	
CE316	How many times did you receive antenatal care when you were pregnant with (name)?<br>**ھ ء ن حعل دوران ي تڪي دفعا طھ معاانو مايو؟**<br>جب آپ (نهم) ک سهیھ حهمله یھ یو حمل ک دو ان آپ ےکتھ مربیہ اپنھ معهئنہ کروایھ؟	Number of times: معهئنوں ک یعداد_______<br>Don't knowمعلوم نہ ہی 98	
CE316 a	What health problems you faced in you **first three** months of pregnancy?<br><br>**توهاني شروعا ت مهيت ۾ صحت حواي سان چا مئنل يا؟**	None---00<br>High blood pressureرھ ءبلڊ پري يس.......... 01<br>Gestational diabetes حمل وا ي شگر ی<br>بيمھ ي ...02<br>Preeclampsia حمل دو ان وڌيل بلڊ<br>پري يس ....03<br>kidney problems گڙدن جھ مسئل................ 04<br>Severe headaches شديد مهِ جو سو............. 05<br>Changes in vision, including temporary loss of vision, blurred vision or light sensitivity نظر جھ<br>مسئل (انک ین اگیهن ڏنڌ،نظرگھٹ ٹ ی ٹں، وشھ سهن<br>(حسهسيت ......06<br>Upper abdominal pain, usually under your ribs on the right side پيٹ ی مم ۑجي ۾ سو ٹ ی ٹی<br>07...گھٹو ڪري سهی پھس پهساٹ ين ۾	Multiple response s جيي مسئل ٺيھ س سب ءٺک ڪيو

		Nausea or vomiting متل يھ الھ ...08 Decreased urine output پيشھب گھٽ اچڻ ...09 Decreased levels of platelets in your blood خون ۾ خون سڪھڻ وا ن جزن ی گھٽ سطح/مقدا رجڻ ....10<br>Impaired liver function ءجگر جوگھٽ ڪم ڪرڻ.11<br>Shortness of breath, caused by fluid in your lungs سھھ ی گھٽتھی/سھڪ ٿ ی ٿں ڦڦڙن ۾ پھ ڻی پرجڻ ی ڪري....12<br>Sudden weight gain and swelling (edema) — particularly in your face and hand اچھنڪ وزن وڌجڻ ۽ سوج ٿ ی ٿں خھص ڪري رٽ پ ير سجڻ 14.... ....13<br>Preterm labor وقتکھن اڳ ۾ ويم جھ سو ٿ ی ٿں<br>Miscarriage حمل ذايعھ ٿ یڻ ....15<br>.17 Anemia ت يکوٽ /ڪمزو ي ....16<br>Infection... متغدي مرض/انفيقشن<br>Breech position ايتو رجڻ بھ ....18 High grade fever ی بخھ اچڻ ...19<br>Other ٻيو ڪجھ 96	
CE316 b	**How did the issue get resolved?**<br>**ينٽ حل يا اي مئلل؟**	Seek care at private hospital سکھ ي اسپتھل مھن گ علج....01<br>Seek care at public hospital خھی اسپتھل مھن علج......02<br>Seek care from NGO hospital خ ٿی اسپتھل مھن علج...03<br>Resolved by its own پھٹھ حل ی ويو...................04<br>Took medication from pharmacy استو يھن دوائون و ييون....05<br>Took home remedies گھريلو نسخو و يو ....06 Usually we don't visit hospital عھم او ٿں سھن اسپتھل ی ويندا آريون...07<br>Seek care from other health provider (homeopathic, herbal) ڪنھن عطھی ۽ اڪر سھن علج و تس.......08<br>Get religious help مذرھ مدد و تی ....09<br>Other ٻيو ڪجھ 96	Multiple responses جيي حل و يھ س سب ءڻڪ ڪيو
CE316 c	What health problems you faced in your **middle three (second trimester)** months of pregnancy?<br>**توھاني وچ ڙٽ مھيٽ ۾ صحت حواي سان چا مئلل يا؟**	None--00<br>High blood pressure رھ ءبلب پري يس.......... 01<br>Gestational diabetes حمل وا ي شگر ی بيمھ ي. ...02<br>Preeclampsia حمل دو ان وڌيل بلڊ پري يس ....03<br>kidney problems گڙدن جھ مسئل................ 04<br>Severe headaches شديد مھ جو سو.............05<br>Changes in vision, including temporary loss of vision, blurred vision or light sensitivity نظر جھ مسئل (انک ين اگيھن ڌنڌ,نظرگھٽ ٿ ی ٿں، وشھ سھن حسھسيت) ......06<br>Upper abdominal pain, usually under your ribs on the right side پيٽ ی مم ۽ ٺجي ۾ سو ٿ یڻ گھٹو ڪري سھی پھس پھسات ين ۾...07<br>Nausea or vomiting متل يھ الھ ...08 Decreased urine output پيشھب گھٽ اچڻ ...09 Decreased levels of platelets in your blood خون ۾ خون سڪھڻ وا ن جزن ی گھٽ	Multiple responses جيي مسئل ٿيھ س سب ءڻڪ ڪيو

		سطح/مقدا رجڻ ....10<br>Impaired liver function ءجگر جوگهٽ ڪم<br>.ڪرڻ11<br>Shortness of breath, caused by fluid in your<br>سھھ ی گھٽنھی/سھک ٹ ی ٹ ڦڦڙن ۾ پھ ٹی پرجڻ ی lungs<br>ڪري....12<br>Sudden weight gain and swelling (edema) — particularly in your face and hand اچھنک وزن<br>13.... وڌجڻ ۽ سوج ٹ ی ٹ خھص ڪري رٹ پ یر سجڻ 14....<br>وقتکھن اگ ۾ ویم جھ سو ٹ ی ٹ Preterm labor<br>Miscarriageٹی حمل ذایعھ ٹ ی....15<br>.17 Anemia ت یکوٹ /ڪمزو ي ....16<br>متغدي مرض/انفیقشن ...Infection<br>Breech position پھ ایتو رجڻ....18 High grade feverاچڻ ی پخھ...19<br>Other ٻيو ڪجھ...96	
CE316 d	How did the issue get resolved?<br>**ینٹ حل یا اي مئل؟**	Seek care at private hospital سڪھ ي اسپتھل مھن گ<br>علج....01<br>Seek care at public hospital خھی اسپتھل مھن<br>علج......02<br>Seek care from NGO hospital خی ٹاسپتھل مھن<br>علج...03<br>Resolved by its own پھٹھ حل ی<br>ویو..................04<br>Took medication from pharmacy اسٹو یھن<br>دوائون و ییون....05<br>Took home remedies گھریلو نسخو و یو....06 Usually we don't visit hospital عھم او ٹیاسھن<br>اسپتھل ی ویندا آریون...07<br>Seek care from other health provider (homeopathic, herbal) ڪنھن عطھی ڈاڪر سھن<br>علج و تس.......08<br>Get religious helpی مذرھ مدد و ....09<br>Otherٻيو ڪجھ 96	Multiple responses جيي حل و پھ س سب ءٹک کیو
CE316 e	What health problems you faced in your **last three** (third trimester) months of pregnancy?<br>**توھاني آخمي مھیٹ ۾ صحت حواي سان چا مئل یا؟**	None---00<br>High blood pressureیس رھ ءبلڊ پري.......... 01<br>Gestational diabetes حمل وا ي شگر ی<br>بیمھ ي. ...02<br>Preeclampsia حمل دو ان وڌیل بلڊ<br>پري یس ....03<br>kidney problems گرڙدن جھ مسئل................ 04<br>Severe headaches شدید مھ جو سو.............. 05<br>Changes in vision, including temporary loss of vision, blurred vision or light sensitivity نظر جھ<br>مسئل (انک ین اگیھن ڌنڌ،نظرگھٽ ٹ ی ٹ، وشھ سھن<br>(حسھسیت ......06<br>Upper abdominal pain, usually under your ribs on the right sideٹی پیٹ ی مم ۾ یجي سو ٹ ی<br>گھٹو ڪري سھی پھس پھسات ین ۾...07<br>Nausea or vomiting متل یھ الھ ...08 Decreased urine output پیشھب گھٹ اچڻ ...09 Decreased levels of platelets in your<br>blood خون ۾ خون سڪھئڻ وا ن جزن ی گھٹ<br>سطح/مقدا رجڻ ....10<br>Impaired liver function ءجگر جوگهٽ ڪم<br>.ڪرڻ11<br>Shortness of breath, caused by fluid in your	Multiple responses جيي مسئل ٹیھ س سب ءٹک کیو

		سهھ ی گھنتهی/سهک ٹ ی ٹں ففڙن ۾ پهِ ئی پرجڻ ی lungs<br>کري....12<br>Sudden weight gain and swelling (edema) — particularly in your face and hand اچھنک وزن<br>13.... وڏجڻ ۽ سوج ٹ ی ٹں خھص کري رٹ پ ير سجڻ 14....<br>وقتکهن اگ ۾ ويم جهِ سو ٹ ی ٹں Preterm labor<br>Miscarriageٹ ئي حمل ذايعهِ ....15<br>.17 Anemia ت يکوٹ /ڪمزو ي ....16<br>متغدي مرض/انفيقشن ... Infection<br>Breech position ...ايتو رجڻ پهِ 18 High grade feverاچڻ یپخهِ ...19<br>Other پيو ڪجهِ 96	
CE316 f	How did the issue get resolved?<br>**ينٹ حل يا اي مئئل؟**	Seek care at private hospital سکهِ ي اسپتهل مهن گ علج....01<br>Seek care at public hospital خهی اسپتهل مهن علج......02<br>Seek care from NGO hospital خ ئراسپتهل مهن علج...03<br>Resolved by its own پهٹهِ حل ی ويو………………04<br>Took medication from pharmacy استو يهن دوائون و ييون....05<br>Took home remedies گھريلو نسخو و يو ....06 Usually we don't visit hospital عهم او ئیسهن اسپتهل ۾ ويندا آريون...07<br>Seek care from other health provider (homeopathic, herbal) ڪنهن عطهی ۽اکر سهن علج و تس.......08<br>Get religious help مذرهِ مدد و ئی ....09<br>Otherپيو ڪجهِ 96	Multiple responses جيي حل و يهِ س سب ءئک کيو
CE317	Did your husband accompany you to any of the antenatal visits?<br>**چا توهان جو مڙس نهن طهِ معااهِ دوران توهان سانگڏ هليو؟**<br>کيهِ آپکهِ شوہرحمل ک ئ معهئنه م يں آپ ک سهيهِ آيهِ يههِ ؟	Yes ہہں 01<br>No نہ يں 02	~~SKIP IF CE206 IS NOT 1~~
CE318	Did anyone (else) accompany you to any of the antenatal visits?<br>**چا بيو و يی هليو توهان سان گڏ حعل نهن ۽ طهِ معااهِ دوران؟**<br>کيهکوی او حمل ک ئ معهے م يں آپ ک ہمراہ يههِ/يهِ؟	Yes ہہں 01<br>No نہ يں 02	IF 2 GO TO CE320
CE319	Who accompanied you to the antenatal visits?<br>**حعل معا دوران بيو ڙکگڏ هئيو؟**<br>حمل ک معهے م يں کون آپ ک سهيهِ يههِ/يهِ؟<br>Select all that are mentioned<br>**.سچا جوابَ ہڏو ۽ لکو**<br>-يمهم جوابهت د ج کريں	Husbandشو ہر 01<br>Other male family member (specify)<br>(فيمل/خهندانکهِ دوسا مرد(وضهحت کريں 02<br>Female family member (specify)<br>(فيمل/خهندان ک عو ت (وضهحت کريں 03<br>Other (specify) ديگر وضهحت کريں 96	
CE320	As part of your antenatal care during this pregnancy, were any of the following done at least once:<br>**هن حعل دوران طهِ معااهِ ۾ هن لئٹ جهِ يی هک دفعا يی وٹيئٹ/ععل يو؟**<br>آپ ک اس (موجودہ) حمل ک دو ان اهِ معهئنه ک وقتکيهِ مند جه ذيل م يں س کوی عمل کم ازکم ايک دفعه کيهِ يههِ؟	Blood pressure بلڈ پری يس (Yes/No/DK)<br>Urine sample پيشهب کهنمونه (Yes/No/DK)<br>Blood sample خون کهِ نمونه (Yes/No/DK)<br>Ultrasound ال ٹراسهئونڈ ------------------ (Yes/No/DK)<br>Received counselling on nutrition<br>غذای مشو ے(Yes/No/DK) ------<br>Received counselling on breastfeeding<br>مهں کهِ دودهِ پلےکهِ مشو ہ(Yes/No/DK)------<br>Received counselling on family planning<br>خهندای مئےوبه يندیکهِ مشو ہ(Yes/No/DK)------<br>Received counselling on danger signs quick follow up if experience any danger sign	

		خطرنھک علم تن ی مشو و(Yes/No/DK)<br>None of the above<br>ان م یں س کوی نہ یں(Yes/No)------<br>Other (Please Specify)<br>دیگر وضھحت کریں-----------------	
CE321	When you were pregnant with (name), did you receive any injection in the arm or shoulder to prevent the baby from getting tetanus, that is convulsions after birth?<br>چھ رن حمل ۾ پورھن تشنج(ٽيٽرس) سھن بچھوء جھ ٽکھ رٿھيھ؟ جب آپ حمل س یھ یوکیھ آپ ے بھزو پر تشنج س بچھؤ ک ٹیک لگوای یھے؟	Yes ہیھں 01<br>98 No نہ یں 02<br>معلوم نہ یں DK	IF 2 go to CE323
CE322	How many times did you receive this tetanus injection during your pregnancy with (name)?<br>هن حعل ۾ توهان تشنج سان بچاو جا ي ٽڪا هٿايا؟<br>آپ ے دو ان حمل کتھ مرتبہ تشنج ک یمھ ی س بچھؤ وال ٹیک لگوای یھے؟	Number of times ٹیکوں ک یعداد __<br>DK معلوم نہ یں 98	
CE323	During this pregnancy, were you given or did you buy any iron tablets, iron syrup, or iron and folic acid supplements?<br>هن حعل ۾ فولد جون گوريون يا شربت، فولڪ ايئڊ جون شيون (توهان اٿ ورتيون يا مم ز تان مليون چا توهان اي ڪاڏيون؟<br>اس حمل ک دو ان کیھ آپ کو فولدک گولیھں / یسبت یھ فولد /فولک ایسڈک اشیھء مل یھ یھ آپ ے خود خریدی یھ؟<br>گولیھں/ یسبت دیکھھی یں show tablets/syrup	Yes ہیھں 01<br>98 No نہ یں 02<br>معلوم نہ یں DK	
CE324	During this pregnancy, did you take any drug for intestinal worms<br>چا هن حعل دوران توهان ا پيٽ جا ڙ يڪا مارڻ واري دواکاڌي هھ؟<br>کیھ اس حمل ک دو ان آپ ے پیٹ ک ک ی ڑروں ک کوی دوا استعمھلک یں؟	Yes ہیھں 01<br>98 No نہ یں 02<br>معلوم نہ یں DK	
Now I would like to ask you about the time when you gave birth to (NAME)<br>-ایھ م یں آپ س (نھم) ک پیدااش س متعلق سوالت پوچھوں ک<br>.را ي مان توهان سان بار پيدائش وقت متعلق جھ سوال اڇندس			
CE 325a	Did you know the expected date of delivery for (NAME)?<br>چا توهاڻ (نام) انداڻ /متوقع تاريييخ خڪ هھ؟<br>کیھ آپکو(نھم) ک متوقع یھ ییییخ پیدااش معلوم یھ؟	Yes ہیھں 01<br>No نہ یں 02	
CE325b	Did you deliver in the same date of EDD or after/before?<br>چا توهان دليوري متوقع تاريييخ ت ي يا يو ء/پهرين ي ؟(لکو ي ٽڪا (ڏينهن م يھ ياگٽ هئا ء توهان چا يو ان بابت؟	Same date..........01<br>After EDD............02<br>Before EDD ....03<br>DK معلوم نہ یں 98	
CE325	Where did you deliver?<br>بار يھ جاو؟<br>(نھم) ک پیدااشکھیں ہوی یھ ؟<br>Only one answer allowed. Probe to identify the type of source. Circle code for source at right.<br>ا يي هڪ جواب چونڊيو .<br>رف ایک جوابکھ انتخھب کریں. اندازہ لگھے ک ل ے مزید یھیل ہوچھں. ذ یعہ ک ل ے دائ یں ارف دئ ے گےکوڈ پر دائرہ لگھئ یں	At home گھر پر 01<br>On the way to facility مرکز صحت جھ تےھوے اس تےے م یں 02<br>**Public health sector**<br>Government hospital (THQ Matiari, Hala ,Saeedabad) سکھ ی ہسپتھل 11<br>13 RHC/MCH آ ایچ س/ایم س ایچ 12<br>BHU/FWC ی ایچ یو/ایف ڈبلو س<br>Other public (specify) دیگر وضھحت 16<br>**Private health sector**<br>Private hospital/clinic ی ہسپتھل/کلینک 21<br>Other private (specify) دیگر وضھحت 26<br>AKHSP facility اے ک ایچ ایس یکھ مرکز صحت 31<br>Other (specify) دیگر وضھحت کریں 96	01 CE326<br>02 CE326<br>11 CE327<br>12 CE327<br>13 CE327<br>16 CE327<br>21 CE327<br>26 CE327<br>31 CE327<br>96 CE327
CE326	Why didn't you deliver (name) in a health facility?<br>توهان اسپتال / صحت مم ز ۾ ويم جو ن مايو؟<br>آپ ے (نھم) ک پیدااشکئ مرکز صحت پرکیوں نہ یں کروا ی ؟	Cost too much خرچہ یہت زیھدہ 01<br>Facility not open مرکزصحتکھل نہ یں 02<br>Too far یہت دو 03<br>Don't trust facility مرکز صحت پر اعتمھد نہ یں 04	

		No female provider at facility مرکز صحت پر<br>دیکھ یبھل ک ل ے کوی عو ت موجود نہ یں 05<br>Husband/family did not allow شوہر/فیمل ے<br>اجھزت نہ یں دی 06<br>07 مرکز صحت پر جھنھ رو ی نہ یں Not necessary<br>Not customary واج نہ ہی 08<br>No transportation ٹرانسپو ٹ نہ ہی 09<br>صحت ک سہولیھتکھ خراب Poor quality service<br>11 معیھ 10<br>ڈیلیو ی یری س ہوی Rapid deliver<br>Other (specify) دیگر وضھحت کریں 96	
CE327	Who assisted with the delivery of (name)?<br>ويم نهن مايو؟<br>نھم) ک پیدااش ک دو انکس ے مدد کرای یھ؟)<br>Ask: "anyone else?"<br>مزید پوچھ یں۔کوی او شخص<br>Record all responses. Probe to determine the type of person. If the respondent says that no one assisted, probe to determine whether any adult was present<br>هيوڳڏ زڪ هئو؟ (معلوم مڻ وشش يو) سچا جواب نوٽ<br>ريو. اگر جواب ڏڏيندڙ چوي ٿي نهن مدد ٿي ما ٿي اچو<br>گھرم زڪ وڏو هئيو؟ يمهم<br>جوابھت یکھ ڈکریں،زچی م یں مدد کرانے والونکھ معلوم کرے ک ل ے<br>پوچھ یں ۔<br>اگر جواب دہندہ کہ تھ ےکہ کئ ے یھ مدد نہ یں کروای یو پوچھ یں<br>آیھکوی بھلغ شخص موجود یھھ<br><br>Yes, ہںھ 01<br>No نہ یں 02<br><br>If any option other than 21 is selected then go to cb10	**Health professional**<br>Doctor ڈای ٹ 11<br>Nurse نرس 12<br>Midwife مڈ وائف 13<br>LHV ایل ایچ وی 14<br>**Other persons**<br>Traditional birth attendant/ Dai دای 21<br>CMW (skilled) کمیون ٹھ مڈ وائف 22<br>Lady health worker لیڈی ہیلتھ و کر 23<br>Relative/friend شتہ دا /دوست 24<br>Self/nobody خود/کوی موجود نہ یں 25<br>Other (specify) دیگر وضھحت کریں 96<br>Don't know معلوم نہ ہی 98	If 21,24 is selected go to CE328
CE328	What are the reasons you preferred a (traditional birth attendant/relative) in the birth of (name)?<br>اها يهڙي وجي هھ جو توهان دا /دوستي تمجيع ڏ ؟<br>وہ کیھ وجوہھت یھ یں جس ک بھعث آپ ے (نھم) ک پیدااش ک وقت مدد ک ل ے<br>دای/دوست کو یرجیح دی؟	Distance فھصلہ 01<br>Better care than facility مرکز صحت ک مقھیل م ہی<br>یہ تر دیکھ یھھل 02<br>Religious reasons مذہبھ وجوہھت 03<br>Husband/family preference شوہر/فیمل ک<br>یرجیح 04<br>Privacy پردہ 05<br>Lower costs کم خرچہ 06<br>Other (specify) دیگر وضھحت کریں 96	
CE 328 -101	Did health care provider use partograph?	Yes…1<br>No…2<br>Don't know…98	
CE 328a	How was (NAME) delivered?<br>ٻار يهڙي رس ٿھ جااو؟<br>نھم) ک پیدااشکس ارح ہوی یھ؟	Normal Vaginal Delivery (NVD)….1<br>نھ مل زچی(NVD)<br>Caesarian Section (C-Section)….2<br>آپریشن س (C-Section)<br>Other (Please Specify)<br>دیگر وضھحت کریں----------------	If 1 selected CE 328a1<br><br>If 2 go to CE 328a2
CE 328a1	Did normal delivery occur in spite of C-section?	Yes …1<br>No…2	If 2 go to reason CE328 a2
CE328a 2	Reasons	Personal preference….1<br>Mother in law preference…2<br>Husband suggested …….3<br>Cultural belief to have normal delivery…4	Multiple options

		There was no complication …5<br>Health care provider advised ……6<br>Others……… 98	
CE 328 a3	Did C-section occur in spite normal delivery?	Yes ….1<br>No……. 2	If 2 go to reason CE 328a4
CE 328 a4	Reasons:	Personal preference….1<br>Mother in law preference…2<br>Husband suggested …….3<br>C- section preference due to lesser labor pain…. 4<br>There was complication…… 5<br>Health care provider advised……. 6<br>Others………98	Multiple options
CE 328 -102	Type of current pregnancy	Single….1<br>Multiple…..2	
CE328b	Was baby moving in womb at the time of labour pains?<br>ڇا ٻار پيٽ ۾ چريو جڏهن ويم جا سور يا ؟ معلوم<br>مڻ وشش يوءِ لکوٽي ما ٻار چرپم آخمي دفع)<br>(ڏهن محئوس هه؟	yes ---ى<br>No ---ى<br>Don't know 98---------------------------------------<br>---------	
CE 328 b1	Was baby moving in womb when you were in hospital?	yes--- ى<br>No ---ى<br>Don't know …. 98	
CE328c	How long were your labour pains?<br>ويم جا سور ي تکی ديم يا/هليا؟	Less than or equal to 30 minute 30 منٽ يهگهٽ Less than or equal to 1 hour1 کلک يهگهٽ---Within of Equal to 12 hours 12 -- کلک يهیٔ ويهه<br>گهٽ ----<br>More than 12 hours (specify)12 ---کلکن کهن<br>(مهِ (کي ترا کلک)	
CE 328 -d1	How much time did it take to decide for seeking care after labour pain/ or any complication started/actual delivery? (within home)	HH [_] [_] or MM [_] [_]<br><br>Not applicable………<br>Don't know ………………….. 98	
CE328d	How much time did it take in heading out for Health facility after labour pain/ or any complication started/actual delivery? (from home to first hospital)<br>توهانی ي تکی ديم ل يل سور/ويم نهن مئی شروع ڙ يٽ کان<br>پوءِ صحت مم ز وچڻ فيصي مڻ ۾؟	HH [_] [_] or MM [_] [_]<br><br>Not applicable………<br>Don't know ………………….. 98	
CE328 d2	How much time did it take in Health facility to receive the care? (Time duration within first hospital)	HH [_] [_] or MM [_] [_]<br><br>Referred out….1<br>Not applicable………2<br><br>Don't know ………………….. 98	
CE328d i	Try to write reason for delay.<br>وجه ٻڌو ءِ لکو.	Reasons for Delay …………………………………..<br>---	User input

		---	(words limit 150)
CE 328e	What health problems you faced at time of delivery?<br>توهاني ويم دوران يهڙا صحت حواي سان مئئل يا؟	Labor that does not progress….01<br>Vaginal discharge ....02<br>Problems with the umbilical cord .....03<br>Abnormal heart rate of the baby.....04<br>Water breaking early ....05<br>Birth asphyxia .... 06<br>Any birth injury........ 07<br>Excessive bleeding .... 08<br>Green/brown water leaking…09<br>Other 96	
CE 328f	How did the issue get resolved?<br>حل يا اي مئئل؟) ينث حل يا اي مئئل؟)	Seek care at private hospital سکهـ ي اسپتهل مهن علج....01<br>Seek care at public hospital گ خهی اسپتهل مهن علج......02<br>Seek care from NGO hospital خ ڀڻ اسپتهل مهن علج...03<br>Resolved by its own پهڻهـ حل ی ويو.................04<br>Took medication from pharmacy استو يهن دوائون و ييون....05<br>Took home remedies گهريلو نسخو و يو ….06 Usually we don’t visit hospital عهم او ڻی اسهن اسپتهل ی ويندا آريون...07<br>Seek care from other health provider (homeopathic, herbal) کنهن عطهی ڊاکر سهن علج و تس.......08<br>Get religious help ڻی مذرهـ مدد و ....09<br>Other ٻيو ڪجهـ 96	
CE 328 f1	Did you had health check up after delivery?	Within 24 hrs…1<br>Within 48 hrs…2<br>After 48 hrs…3<br>Didn’t have health check up	
CE 328g	What health problems you faced within one week after delivery?<br>توهاني بار جعڻ کان پوءِ پيهرين هف ته ۾ يهڙا صحت هواي سان مئئل يا؟	Infection or sepsis..........................01<br>Excessive bleeding after giving birth (hemorrhage) ....02<br>Heavy vaginal discharge ....03<br>Depression or mental stress ....04<br>A painful, hard, warm, red area (usually only on one breast) ....05<br>Fever, chills, muscle aches ....06<br>Fatigue, or a headache ....07<br>Other 96	
CE 328h	How did the issue get resolved?<br>(ينث حل يا اي مئئل؟ (حل يا اي مئئل؟	Seek care at private hospital سکهـ ي اسپتهل مهن علج....01<br>Seek care at public hospital گ خهی اسپتهل مهن علج......02<br>Seek care from NGO hospital خ ڀڻ اسپتهل مهن علج...03<br>Resolved by its own پهڻهـ حل ی ويو.................04<br>Took medication from pharmacy استو يهن دوائون و ييون....05<br>Took home remedies گهريلو نسخو و يو ….06	

		Usually we don't visit hospital عهم اوی سهن<br>07...اسپتهل ی ویندا آریون<br>Seek care from other health provider (homeopathic, herbal) کنهن عطهی ڈاکٹر سهن<br>علج و تس.......08<br>Get religious help مذرهہ مدد وٹی ....09<br>Other بیو کجھ 96	
CE329	Did you or your family make any advance preparations for the birth of (name) while you/she were pregnant?<br>جڏهن توهان حعل سان هئا تڏهن توهان يا توهان فيعي ويم حواي سان ي تياريون يون؟ جب اپ<br>حمل س یھ ین یوکیھ اپ یھ اپ ک فیمل ے (نهم) ک پیدااش<br>ک ل ے پہل س کوی بیھ ی ک یھ؟	Yes بہں 01<br>No نہ یں 02<br>Don't know 98	If 2 go to CE331
CE330	Which of the following preparations did you/she make?<br>يهڙيون تياريون يون توهان؟	Place of delivery زچگی ک ج (Yes/No)<br>Who delivers زچیکون کراے گھ (Yes/No)<br>Set aside funds فنڈز /پیسوں کھ انتظهم (Yes/No) Alternate funds قم متبهدل<br>Transport ٹرانسپو ٹ (Yes/No)<br>Blood donor خون عطیھکر ے وال (Yes/No)<br>Danger signs during pregnancy ک خطرنهک علمهت حمل (Yes/No)<br>Danger signs during delivery خطرنهک علمهت (Yes/No)<br>Accompaniment عو ت ک ہمراہ کوی شخص (Yes/No)	
CE331	During your pregnancy with (name) did you receive any of the following types of support from your husband?<br>هن حعل دوران توهان مڙس يهڙي مدد ه توهان ؟<br>اس حمل ک دو ان اپ ک شوہرک ارف سکیھ مند جه ذیلکھموں م یں سکئ کھم م یں کو ی مدد مل ؟	Ensure accompaniment ANC اھ معهئے کراے ک ل ے اپ کو لکر چل یں (Yes/No/DK)<br>Ensure accompaniment delivery زچگی ک وقت جھے ہوے آپ ک سھیھ چل یں (Yes/No/DK)<br>Purchased items چ یں خریدیں (Yes/No/DK)<br>Domestic tasks گھریلوکھم کھج (Yes/No/DK) Child care بچوں ک دیکھ یھهل (Yes/No/DK)<br>Encouraged pre-delivery visits (ANC) حمل ک دو ان اھ معهئے کراے ک حوصله افزای(Yes/No/DK)	If even any one No go to CE332
CE332	If not, what was the primary reason why you did not receive support during pregnancy?<br>اگر ني ي يهڙي بنيادي وجي هھ حعل دوران مدد ني ملڻ ؟<br>اگر نہ یں یوکیھ یریھدی وجه یھ کھ آپکو دو ان حمل مدد نہ یں مل ؟<br>Select all applicable<br>یمهم ممکنه جوابهت لکھ یں<br>Probe: any other reasons?<br>ا اڻ لکيل وجھ ؟ٻڌو ۽ لکو .<br>مزید پوچھ یں :کوی او وجوبهت؟	Husband/male family member was away<br>/خهندان کھ مرد کن دو یھھ شوہر<br>Childbirth/pregnancy is not for men<br>پیدااش /حمل مردوں ک ل ے نہ یں ....2<br>Husband/male family member do not perceive this as important<br>شوہر/فیمل ک مرد اس کو اہم نہ یں سمجھ ے ہ یں 3<br>I have other female family members that provide support م یرے پهس فیمل ک دوسی عو ی یں موجود ہ یں جو مدد فراہم کر تی ہ یں ....4<br>I have my own finances م یرےپهس اپنھ پیسه /آمدی ے...............5<br>I do not require support/help مجھے مددک رو ت نہ یں ے ....6<br>Other (specify) دیگر وضهحت کریں_________________96	
CE333	What is the source of financial support for your maternal, newborn and child health related needs?<br>توهان جو ماي امداد جو يهڙو ذريعو آي ماِ ۽ ٻار صحت خرچ حواي سان؟<br>س متعلقه رو ت کو (MNCH) آپ ک پهس مهں او ے ک صحت پو اکرے ک ل ے مهل امداد ککیھ ذ ائع موجود ہ یں؟	Family (husband or own resource)<br>شوہر یھ اپ وسهئل) خهندان)<br>.....................................01<br>Bank/Loan from organization<br>ینک/کئ ادا ے س قرض<br>.....................................02<br>Community health revolving fund	

		کمیون ٹھ ہیلتھ یوالوننگ فنڈ<br>..03<br>Borrowing from relative/friend<br>دوست / شته دا س ادرھ پیسه<br>..................................04<br>Selling assets اٹھئوں ک فروخت<br>.............................05<br>None needed کئ یھ چ یرک روت نه یں<br>.....................06<br>Other (specify) دیگر وضھحت<br>کریں_______________96	
CE334	For what purpose was the majority of this financial support utilized?<br>**صحت ل ۽ ورتل/جعع یل ماي امداد جوگگٹو حصو نهن مقصد ل ۽ خرچ یو؟**<br>مھل امدادکھ زیھده یرحےبکس مۂد ک ل ے استعمھل کیھگیھ ؟	Transport نقل وحرکت 01<br>Pre-delivery (Antenatal) care دو ان حمل اھ گ معھئنه 02<br>Delivery/C-section زچی/یڑا آپریشن 03<br>Emergency ایمرجزئ 04<br>After-delivery (Post natal) care زچی ک بعدکھ - گ دیکھ یھھل 05<br>Neonatal or under 5 child health نومولود یھ 5 سھل س کم عمر ے ک صحت 06<br>Other (specify) دیگر وضھحت<br>کریں_______________96	
CE334-101	Time duration between first health facility access and death (duration)	HH [_] [_] or MM [_] [_]<br><br>Not applicable.........<br>Don't know 98	
CE334a	Did you or deceased get referred out from facilities?<br>**چا توهاني اسپتال ڏانهن مو ليو ويو؟**	Yes ....1<br>No..............................2<br>Don't know 98	If 2 and 98 go to CE334e
CE334b	How many times you or deceased got referred out?<br>**ي تکی دفع اسپتالون بدلیون توهان ؟**	Numbers________________________	
CE334c	What did facility HCPs tell you or deceased on each referral out?<br>**هم دفعا اسپتال بدلاڻ وقت صحت ار ٿ توهاني چا چھ مو ليو يا توهان چو ويا؟**	Facility not open اسپتھل یند رھ------------------- 01<br>Absence of facility staff اسپتھل ۾ صحت کھ کن موجود ن رئھ--- 02<br>Absence of required medications رو ت جون دوائون ن رئيون.-- 03<br>Patient did not satisfy and preferred to go 04 elsewhere مریض مطمس ن رئو ۽ ہیو کھ علج ل ءء ی ویو ---<br>Unbearable expenses خرچ یرداشت نھ کري سگھیھ---05<br>Because staff had insufficient time for evaluating patient thoroughly وقت ن رئو مریض ک یو و جھچڻ ل ءء--------------------------06<br>For investigation test, diagnosis, procedure etc. کنھن میڊیکل وجی سھن (ٹیسٽون، آپریشن، بیمھ ي --------------- ی سجھٹپ وغ یرا ل ءء) --------07<br>Type of care availability on facility mismatch with complication اسپتھل ی نوبعیت مسئل ی حل ------------ (جی تري ن رھ (نندي اسپتھل رھ ---------08<br>Other (specify) (ہیو کجھ (بڏایو-------------------96	
CE334d	In how much time they referred out (you or deceased out)?<br>**توهاني ريفم مڻ ۾ يکی ديم لڳ هم دفعا؟ ي**	HH [_] [_] or MM [_] [_]<br><br>Not applicable........01	

		Don’t know 98	
CE334e	What was the situation when (NAME) was dying?<br>**(NAME) (ممڻ وقت چا حالت ههڃ؟(وضاحت سان لکو**<br>**(no read outs)**	1. unresolved bleeding<br>2. unconscious<br>3. unresolved fits<br>4. unresolved fever and infection<br>5. unresolved sever pain<br>6. breathing difficulty/gasping<br>7. untreated retained conception products<br>8. Cephalopelvic Disproportion<br>9. obstructed labour<br>10. prolonged labour<br>11. unresolved chest infection<br>12. fluid aspiration<br>13. congenital anomaly<br>14. prematurity<br>15. low birth weight<br>16. hypoglycemia<br>17. hypothermia<br>18. hypoxia<br>19. birth asphyxia<br>20. birth trauma<br>21. hydrocephalus<br>22. no fetal movement /no fetal heart soundsf<br>23. cord strangulation<br>24. unresolved jaundice<br>25. unresolved diarrhea<br>26. others.....96	multiple
CE 334 f	Was resuscitation given to the mother/neonates	Yes....1<br>NO....2	

Danger Signs Awareness Module (DW) اتا			
CE401	**Are you aware of any danger signs during pregnancy? چا توهاني حعل دوران خطمناڪ تت علم باري ۾ اجاڻ آي؟**	**Yes, ہاں 01**<br>**No.نه ڙت 02**	02 CE403

CE402	Could you please name any danger signs during pregnancy that you are awa re of?<br>براے مہربھیکھ اپ حمل گی دو ان خطرنھک علمھت م یں س کنکھ نھم یتھ سک یں ک؟<br>Select all that are mentioned, DO NOT READ LIST OR PROMPT with any suggestions<br>جواب دہندہ ک یتھے ہوے یمھم جوابھتکھ انتخھب کریں- فہرست نہ پڑر یں نھکئ جواب ک متعلق کوی یجویز دیں<br>Probe: Any other danger signs?<br>پوچھ یں : کوی او خطرنھک علمھت<br>Keep asking for more danger signs until the participant cannot recall any additional signs. Circle all that are mentioned, but do not prompt with any suggestions.<br>جب یککہ سکتکھ ٓاپکو مزید علمھت نہ یں یتھ سکتھ،سوال پوچھ ے ہ یں<br>یمھم جوابھتکھ انتخھب کریں لیکن کئ جواب ک ل ے کوی یجویز نہ دیں-	Difficulty breathing سھنس لیے م یںدشوا ی 01<br>Fatigue/tiredness یھکھو ٹ 02<br>Vaginal bleeding فرج س خون انھ 03<br>Baby not moving ےکھ حرکت نہ کرنھ 04<br>Loss of consciousness ے ہوش ہونھ 05<br>Convulsions جھٹک 06<br>Headache and blurred vision شدید س د د او ےنھی ک مسھئل<br>..07<br>Early labour pain before term وقت س پہل زچیکھ د د گ 08<br>Vaginal discharge فرج س اوبتکھ اخراج 09<br>High blood pressure بلند فشھ خون 10<br>High grade fever (>101) یبخھ .... 11<br>Swelling of face and arms چھرے او بھزو م یںسوجن 12<br>Does not know any of the above مند جہ بھل علمھت م یں س کئ ک بھ ے م یں کوی علم نہ یں 98	
CE403	**Are you aware of any danger signs during labor and childbirth? چا توهاني ويم سورن ۽ بار پيدائش دوران يندڙ خطمناڪ علم تڻ باري ۾ ا جاڻ آي؟**	Yes ہھں .... 01<br>No نہ یں 02	02 CE405
CE404	Could you please name any danger signs during labour and childbirth?<br>کیھ اپ دو ان زچی ک د گ د او ے ک پیدااش ک خطرنھک علمھت م یں س کئ علمتکھ نھم یتھ سک یں گ یں ؟<br>Select all that are mentioned, DO NOT READ LIST OR PROMPT with any suggestions<br>جواب دہندہ ک یتھے گ یمھم جوابھت لکھ یں- لسٹ نہ پڑر یں یھکئ جواب ک متعلق کوی یجویز نہ دیں<br>Probe: Any other danger signs?<br>Keep asking for more danger signs until the participant cannot recall any additional signs. Circle all that are mentioned, but do not prompt with any suggestions.<br>پوچھ یں : کوی او خطرنھ علمھ ات<br>پوچھے ہ یں کہ جب یکئ سکتکھ ٓاپکو مزیدعلمھت نہ یں بھ سکتھ<br>یمھم جوابھت کھ انتخھب کریں لیکنکئ جواب ک ل ے کوی یجویز نہ دیں-	Severe vaginal bleeding (>12 hours)<br>گھن ے س زیھدہ) فر ج س خونکھ یہت زیھدہ یہہ جھنھ (12<br>01<br>Prolonged labour ے ک پیدااشکھ عمل اویل ہونھ .... 02<br>Convulsions جھٹک 03<br>04 Retained placenta پیدااش ک وقت پو ے انول کھ خھ ج نہ یں ہونھ<br>Baby lying sideways or upside down پیدااش ک پوزیشن ایک ارف یھ ال ٹھ ہونھ ....05<br>Fever بخھ 06<br>Green or brown waters سر یھ یھو ے نگ کھ پھی .... 07<br>Headache and blurred vision شدید س د د او ےنھی ک مسھئل ....<br>.........................08<br>Sudden, steady severe pain at the top of the belly پیٹ ک اوپر وال حص م یں اچھنک شدید د د ہونھ ....10<br>High blood pressure بلند فشھ خون .... 12<br>پو ے جسم پر سوجن<br>Does not know any of the above مند جہ بھل علمھت م یں س کئ ک بھ ے م یں کوی علم نہ یں 98	
CE405	**Are you aware of any danger signs after delivery (for yourself) چا توهاني خک آي ويم کان بو ۽ خطمناڪ علم تڻ ؟**	**Yes ہاں 01**<br>**No نہ ڑت. 02**	02 CE407
CE406	What are some of the danger signs that you should watch out for after your delivery?<br>زچی گ ک بعد وہ چندکو ن س خطرنھک علمھت ہ یں جن	High Fever, lower abdominal pain or foul smelling discharge (infection) یر بخھ ، پیٹ ک نیچل حص م یں د دیھ بدیو دا اوبت..01	S

	س اپ کو خردا ہنھ چھ ئ ے ؟<br>Select all that are mentioned, DO NOT READ LIST OR PROMPT with any suggestions<br>جواب دہندہ ک یتھے ہوے یمھم جوابھتکھ انتخھب کریں -<br>فھرست نه پڑر ین نھئ جواب ک متعلقکوی یجویز دیں	Severe headache, blurred vision, high blood pressure<br>شدید س د د ، انکھوں کھ درندل پن ، بلند فشھ خون.............02<br>Convulsions or fits جھٹک/ دو ے ....03<br>Eclampsia حمل ک دو ان بلڈ پری یسکھ یڑھ جھنھ.....................04 گ<br>Heavy vaginal bleeding (PPH) زچی ک 24 گھن ٹے ک اند و بھ<br>خون کھ اخراج..05<br>Urinary or fecal incontinence (obstetric fistula) فل یھ ے<br>پیشھب کوکن ٹرول کرے ک صلحیتکھکمزو ہونھ/<br>ہونھ .... 06<br>Extreme tiredness, Anemia یہت زیھدہ یھکھوٹ یھ خون ککم<br>..07<br>Anxiety or depression (puerperal psychosis پریشھی یھ<br>ذہیھ دبھؤ...08<br>Breast problems (engorgement, sore, cracked bleeding or inverted nipples) چھھ تی ک مسھئل (چھھ تیکھ<br>سخت ہونھ او سوج جھنھ ، زخم یھ خون کھ انھ ، کپتھن کھ اند ک ارف<br>جھنھ ....09<br>Does not know any of the above مند جه بھل علمھت م یں<br>س ئ ک بھ ے م ین کوی علم<br>نه ین...98	
CE407	**Are you aware of any danger signs for newborns?** **چا توھاني نھ جاول ہار**<br>**زکصحتیا خطمناک علم تن اھم ز یٹ خک**<br>**آي؟**	**Yes** **ہاں** **01**<br>**No** **نه ژت.** **02**	2 End of survey<br>سوے کھ اختتھم
CE408	Could you please name any danger signs for newborns?<br>برائے مہربھیکیھ اپ نومولود بچوں ک خطرنھک علمھت م ین سکئ علمتکھ نھم یتھ سک ین گ ین -<br>DO NOT PROMPT<br>پوچھ ین کوی او خطرنھک علمت<br>Probe: Any other danger signs?<br>Keep asking for more danger signs until the participant cannot recall any additional signs. Circle all that are mentioned, but do not prompt with any suggestions.<br>پوچھے تی ین که جب یک سکنکھ اپ کومزید علمھت نه ین بھ سکتھ<br>یمھم جوابھت کھ انتخھب کریں لیکنئ جواب ک ل ے کوی یجویز نه دیں-	Convulsions/spasms/rigidity مروڑ / جھٹک/<br>بغ یر لچک 01<br>Movement only when stimulated or no movement, even when stimulated ی یروی محرک<br>پرکوی حرکت یھ اسٹیمولیٹ پر یھکوی حرکت نه ین 03 02<br>Not feeding well صحیح ارح دودھ نه ی نھ<br>Fever بخھ 04<br>Difficult/fast breathing سھنس لیے م یں دشوا ی /ی ی فتھ<br>سھنس 05<br>Lethargy/unconsciousness سس ت ے ہو ش 06<br>Yellow or pale color on skin eyes جلد / اانکھوں کھ پیل ہونھ ،<br>ز د ہونھ 07<br>Low birth weight پیدااش ک وقت کم وزن 08<br>Not crying بچه و نه ی ں بھ 09<br><br>Does not know any of the above مند جه بھل علمھت م یں<br>س ئ ک بھ ے م ین کوی علم نه ین 98	
CE 409	In your opinion what are the factors contributing to this incident?	Lack of recognizing danger signs during pregnancy and newborn	
		Unnecessary myths and health beliefs	
		Lack of power for decision making of the women present at child birth at household level	
		Fear of being ill-treated at health facility	
		Lack of money to pay for medical expenses and cost of transportation	

		Lack of encouragement from relatives and community members to seek care	
		Reluctance from the mother or the family due to cultural constraints	
		Lack of companion in going to the health facility	
		No available person to take care of the children, the home and livestock	
		Lack of family planning knowledge	
		Preference for female doctors	
		Involvement of other family members in decision making	
		Facility/Dr preference (even if it's far away)	
		Distance of one facility from another (referred by facility)	
		Delay in traveling after identification of problem (self-preference)	
		Lack of community support system	
		Lack of awareness of existing services	
		Lack of roads or poor condition of roads	
		Lack of emergency transportation whether by land	
		Lack of proper referral by LHW and other community health providers	
		No proper documentation of referrals at health facility	
		Weak referral system includes transportation and communication	
		Lack of equipment for emergency	
		Lack of competence of health care providers to deliver EmOC /complicated cases	
		Gender preference of healthcare providers	
		Shortages of emergency medicines	
		Lack of counseling by health care provider	
		Lack of ultrasound machine/ labs at facility	
		Lack of blood transfusion facility	

		Lack of health personnel especially at night	
		Lack of data reporting and recording	
	Remarks (qualitative narratives)		Limit 500 words

Case Notification Form S1 Online Supplementary Document

Identifying delays impacting maternal and perinatal deaths using a facility-based death audit review system integrated with community engagement: A mixed methods study

Case Notification form

S.No	Date of case notification	Case notified by	Name of area LHW	Name of nearest HF	Name of village	Name of UC	Name of taluka	Delivery mode	Place of ANC	Place of delivery	Delivery time	Place of Death	Time of Death	Case type	Date of death/referral case	Number of Referrals	Case Id (as on consent form)	Informer Relation with deceased
